# Lentil, chickpea, microalgae, and duckweed proteins in plant-based foods: a food chemistry perspective on composition, processing, functionality, and digestibility

**DOI:** 10.1016/j.fochx.2026.104744

**Published:** 2026-11-05

**Authors:** Duy H. Truong, Chi Hao Nguyen

**Affiliations:** Institute of Biotechnology and Food Technology, Industrial University of Ho Chi Minh City, 12 Nguyen Van Bao Street, Hanh Thong Ward, Ho Chi Minh City, Viet Nam

**Keywords:** Plant proteins, Food chemistry, Microalgae, Duckweed, Protein functionality, Digestibility, Structure–function

## Abstract

Growing demand for alternative protein ingredients has expanded research on lentil, chickpea, microalgae, and duckweed. Cross-source comparison remains difficult because material form, analytical method, processing history, digestibility model, and functional assay often differ among studies. Quantitative primary evidence was evaluated using a material-method-structure-function-matrix approach. Microalgal nitrogen-to-protein conversion factors ranged from 2.53 to 5.77, indicating limitations of a universal factor of 6.25. Processed lentil and chickpea showed fecal-protein-based DIAAS-type values of 44–58 and 78–84, respectively, whereas cooked chickpea showed true-ileal DIAAS values of 67–76. High-pressure homogenization increased lentil protein solubility while reducing particle size. Hydrolysis of *Wolffia globosa* increased solubility but reduced oil-binding capacity. Evidence remains broader for lentil and chickpea, whereas interpretation of microalgal and duckweed proteins is more condition-dependent. Direct validation in the intended food matrix is therefore more informative than cross-source ranking.

## Highlights

• Nitrogen-to-protein factors ranged from 2.53 to 5.77 in microalgae.
• Protein-quality scores depend strongly on digestibility method and food form.
• Lentil homogenization improved solubility as particle size decreased.
• Duckweed hydrolysis improved solubility but reduced oil-binding capacity.
• Safety and regulation depend on species and final product form.

## 1 Introduction

Consumer demand for innovation in these emerging plant-based categories continues to rise with desirable protein ingredients needed that not only have a stable nutritional profile but also offer reliable functionality. Global retail sales of plant-based meat, seafood, milk, yogurt, ice cream, and cheese reached USD 28.6 billion in 2024, with the U.S. plant-based food market reaching USD 8.1 billion alone (Good Food, 2025). Persistent flavor, texture, and price challenges reflect that protein ingredients cannot be chosen based solely on their protein content or percentage alone. Solubility, water- and oil-binding capacity, emulsification, foaming, gelation, and interaction with other matrix ingredients all affect the structure and sensory properties of formulated foods (Ma et al., 2022).

Proteins from soy and pea plants are already widely used. However, off-flavors, allergenicity, and functional constraints in certain applications continue to drive interest in other plant-protein sources and protein combinations (Jang & Lee, 2024; Lee et al., 2024). Lentil and chickpea are promising candidates since their proteins can contribute to solubility, emulsification, foaming, and gelation. However, protein ingredients vary widely in these properties depending on ingredient form, protein extraction method, pH and thermal exposure history, drying conditions used, and residual non-protein constituents (Aghababaei et al., 2024; Grasso et al., 2022). Their use may also be constrained by beany or green flavors, antinutritional compounds such as protease inhibitors and phytates, allergenicity in susceptible consumers, and processing-induced loss of solubility or colloidal stability (Abu Risha et al., 2024; Malterre et al., 2024; Saffarionpour, 2024).

Seed legumes are compared to microalgae and duckweed here because aquatic-derived proteins present analytical and processing challenges less prominent among pulse ingredients. Lentil and chickpea ingredients are most often studied as seeds, flours, concentrates, or isolates, while microalgal and duckweed research typically reports protein metrics for whole biomass, disrupted cells, protein-enriched extracts, concentrates, or isolates. Four source groups are therefore included here to determine whether published differences in protein quantity, digestibility, or functionality are attributable to source identity or are instead the result of differing material form, cellular structure, non-protein composition, growth environment, protein extraction/recovery methods, and/or analytical approach. Microalgal food-use potential may be strongly affected by cell-wall attributes, growth system, downstream processing, pigment/flavor alteration/removal, and production cost (Ali et al., 2025; Mosibo et al., 2024). Duckweed faces similar hurdles because protein composition and recovery rates can vary based on species, growth conditions, water quality during cultivation, and extraction technique while food-matrix and consumer-validation data are less complete than for established legume proteins (Muller et al., 2025; Sulaiman et al., 2025).

Several reviews have recently summarized protein metrics for these protein sources individually: lentil proteins (Aghababaei et al., 2024), chickpea proteins (Grasso et al., 2022), microalgal proteins (Mosibo et al., 2024), and duckweed proteins (Muller et al., 2025). The body of literature specific to each protein source is sizable, but these reviews do not establish a common basis for determining if apparently disparate protein values were obtained from materially similar ingredient forms, analytical techniques, ingredient-processing histories, digestibility models, functional tests, and food matrices. The primary knowledge-gap addressed here is not insufficient data for any particular protein source, but rather the lack of a common analytical basis for reviewing evidence from distinct protein-source systems.

Based on these principles, this review analyzes lentil, chickpea, microalgal, and duckweed proteins according to a material-method-structure-function-matrix framework. The comparison is structured from material and taxonomic identity to protein determination, conversion of nitrogen-to-protein, amino-acid analysis, digestibility, processing-induced structural changes, techno-functional responses, food-matrix level performance, safety, and regulatory status. The above topics are considered related, but not equivalent: higher protein concentration does not necessarily imply higher digestibility or functionality, in vitro digestion does not necessarily reflect absorption or bioavailability, and isolated measurements of functionality do not necessarily predict formulated food performance without matrix-level evidence. Given the limited number of head-to-head studies comparing all four sources under matched analytical and processing conditions, differences between studies are not used to infer superiority of one protein source over another. Quantitative data can only be compared across sources if the material basis, analytical method, processing conditions and measured endpoints are clearly defined. As such, the objective of this review is not to rank protein sources, but rather to highlight where cross-source comparison is valid, where conclusions are dependent on conditions, or where gaps in analytical or applied research currently limit food-protein design.

## 2 Literature search and review approach

This article was designed as a structured narrative review with comparative analysis, rather than as a systematic review or meta-analysis. The bibliographic search ranged from January 2015 to 12 August 2026 and is up to date as of 12 August 2026. In response to the second-round review, an additional targeted verification search was conducted on 10 October 2026 for ileal amino-acid digestibility and digestible indispensable amino acid score (DIAAS) evidence for cooked lentil and chickpea in growing-pig or rat models. Search terms combined “lentil” or “*Lens culinaris*” and “chickpea” or “*Cicer arietinum*” with “DIAAS”, “digestible indispensable amino acid score”, “true ileal digestibility”, “ileal amino acid digestibility”, “pig”, “rat”, and “cooked”. Older publications were only kept if they offered analytical, methodological, safety, or regulatory basis or background information pertinent to quantifying proteins, amino acids, determining digestibility, controlling contaminants, or interpreting regulations. Regulatory and web-source verification was completed on 14 August 2026. Literature searches were conducted in Scopus, Web of Science, PubMed, ScienceDirect, and Google Scholar. Searches of the European Food Safety Authority (EFSA), European Commission novel-food resources, and U.S. regulatory sources were conducted where applicable when regulatory status or contaminant limits for the protein sources were discussed. Searches included the four protein-source terms (lentil, chickpea, microalgae and duckweed) paired with terms relating to protein composition/extraction, functionality, digestibility, food application, safety, and regulations. Full search strings with Boolean operators for each protein source including cross-cutting analytical searches can be found in Supplementary Table S2.

The retrieved literature was screened using a staged procedure appropriate for a structured narrative review. Titles and abstracts were first examined for relevance to at least one predefined analytical dimension. Potentially relevant full texts were then assessed for protein source or species, ingredient form, material basis, protein-determination method, nitrogen-to-protein conversion factor, amino acid analytical method, digestibility or protein-quality metric, processing or extraction condition, structure-function indicator, techno-functional endpoint, food-matrix application, sensory or safety concern, and regulatory status. Retained sources were classified as primary experimental evidence, review-level background, methodological evidence, or official regulatory/safety evidence.

Studies were retained if they provided information that could inform one or more dimensions of analysis reviewed, including composition, protein determination, amino acid analysis, digestibility or protein quality, extraction or processing, structure-function relationships, techno-functional properties, sensory quality, safety, regulation, or food-matrix application. Quantitative discussion of composition, processing/enrichment conditions, structure-function relationships, digestibility, functionality and application performance was based primarily on original experimental studies. Review articles were generally used only to contextualize larger scale research directions and gaps in knowledge. Evidence from studies conducted solely for animal feed, broiler digestibility, wastewater treatment, bioenergy, agronomy, or environmental remediation was not used as supporting evidence for quality, digestibility, yield, or application performance of food proteins for human use. Similarly, studies conducted primarily to formulate animal feed were not used to establish human-food application performance. Narrowly defined methodological evidence on amino-acid digestibility from animal-feeding studies was the exception if the experimental model and analytical procedures were directly relevant to protein-quality assessment. For example, growing-pig digestibility data were retained as indirect background evidence for standardized ileal amino acid digestibility of steam-pelleted diets containing lentils (Cargo-Froom et al., 2023), but were not considered interchangeable with the digestibility of cooked lentil foods intended for human consumption. Growing-pig ileal digestibility studies were considered more directly relevant to food-protein-quality assessment when the pulses were prepared for human consumption (Han et al., 2020). These studies were not used as direct evidence of application performance when retained for narrowly defined background purposes.

Extracted information per retained study included, where available, protein source/species, ingredient form, basis of material, basis of protein determination/conversion of nitrogen to protein, amino acid analysis method, digestibility/protein-quality assessment method, extraction/processing condition, assay condition, measured structure-function indicators/measured functional endpoint, food matrix, safety concern/regulatory status, and key limitations relevant to comparison across studies. Comparisons were then synthesized across the four protein-source categories, with consideration for whether studies could be compared directly or if comparison could only be made on a condition-dependent basis.

Because this review was not a systematic review or meta-analysis, no Preferred Reporting Items for Systematic Reviews and Meta-Analyses (PRISMA) flow diagram was used, no formal counts of records screened were made, and no risk-of-bias analysis was conducted. The reporting of the methods was intended to offer transparency and included information on the search window, databases searched, full search strings, staged screening methodology, inclusion/exclusion criteria, categories of data extracted, and evidence classification methods. Studies included in Table 1 were selected as representative primary experimental evidence when they clearly defined the protein source or species, ingredient form or processing condition, and at least one measurable endpoint relevant to composition, extraction, structure, digestibility, functionality, or food-matrix performance.

**Table 1:** Representative primary experimental evidence and comparative interpretation for lentil, chickpea, microalgal, and duckweed proteins.

Source/primary study	Material/key condition	Representative quantitative result(s)	Comparative interpretation
Lentil/chickpea – Higa et al. (2024)	Green-lentil and chickpea isolates; AEIP vs SE	Green lentil: IVPD 84.30 ± 1.57 vs 78.42 ± 1.47%; IV-PDCAAS 45.61 ± 0.85 vs 43.64 ± 0.82%. Chickpea: IVPD 81.44 ± 1.08 vs 76.16 ± 0.94%; IV-PDCAAS 57.70 ± 0.76 vs 54.82 ± 0.68%	Differences between AEIP and SE indicate that extraction route contributes to the measured digestibility and protein-quality response; values should therefore not be interpreted as source-specific constants
Lentil – Malterre et al. (2024)	Protein isolate; high-shear mixing	Particle diameter 5.13 ± 0.58 → 1.72 ± 0.01 μm; solubility 46.87 ± 1.55 → 68.42 ± 8.04%; separation rate 71.23 ± 18.15 → 24.16 ± 2.58% h^−1^	Particle disruption was associated with greater solubility and improved dispersion stability under the tested mixing conditions
Lentil – Malterre et al. (2025)	Protein isolate; HPH 0–180 MPa	Particle diameter 10.7 ± 1.1 → 0.33 ± 0.06 μm; surface hydrophobicity 614 ± 66 → 1312 ± 82; solubility 62.5 ± 2.63 → 95.3 ± 0.68%	Reduced particle size and greater surface exposure accompanied improved solubility, but the response was pressure-dependent and not strictly monotonic
Lentil – Nosworthy et al. (2018)	Extruded, cooked and baked red/green lentils	Fecal-protein-based DIAAS-type scores: red lentil 58, 53 and 50; green lentil 53, 49 and 44, respectively	Processing affected the reported protein-quality response, but the fecal-protein-based values are DIAAS-type estimates and should not be interpreted as true-ileal DIAAS
Lentil – Caldeira et al. (2024)	Protein concentrate; formulated croquette	Concentrate contained 85% protein (DW) at 14% mass yield; sensory acceptance was comparable with the commercial clean-taste concentrate	Provides direct translation from protein recovery to a formulated food, although performance remains specific to the tested concentrate and croquette matrix
Lentil/chickpea – Ladjal-Ettoumi et al. (2016)	Protein isolates; pH-dependent emulsification	*d*_4,3_ decreased from 21.2 ± 0.1 to 4.1 ± 0.1 μm for chickpea and from 23.6 ± 1.4 to 4.3 ± 0.2 μm for lentil between pH 4.5 and 8	Strong pH dependence indicates that colloidal behavior is controlled partly by solution conditions rather than botanical identity alone
Mixed plant proteins – De Angelis et al. (2024)	Commercial DF vs WE ingredients	Final *G*′: 2183 ± 986 vs 768 ± 1340 Pa; individual values overlapped markedly (DF 974–4390 Pa; WE 0.11–3563 Pa)	The overlapping ranges and heterogeneous ingredient sets prevent a general rheological advantage from being attributed to fractionation route alone
Chickpea – Gao et al. (2025)	Osborne-fractionated proteins	At pH 7, albumin FC = 70 ± 3% and FS = 41 ± 1%; EC ranged 9.95–18.27 mL/g and ES 48.55–67.14 min among fractions	Fraction-specific functional differences accompanied compositional and structural differences, but multiple variables changed simultaneously and no single mediator was isolated
Chickpea – Soto-Madrid et al. (2023)	Protein fractions; interfacial assays	Protein recovery ≈82 ± 3%; globulin surface hydrophobicity = 30,110 ± 2248 a.u.; interfacial Πeq = 19.70 ± 0.27 mN/m	Fraction-dependent surface characteristics were associated with different interfacial behavior, supporting ingredient-specific rather than source-wide interpretation
Chickpea – Lakshmipathy et al. (2026)	Acid vs alkali extraction	Acid extraction gave 83.45 ± 3.35% purity; LOX decreased 3.27 ± 0.15 → 0.75 ± 0.04 U/g and hexanal 269.4 ± 17.74 → 41.0 ± 1.13 ppb	Extraction conditions affected both protein enrichment and flavor-related chemical markers; lower LOX activity and hexanal are consistent with reduced oxidation-related off-flavor potential
Chickpea – Arshad P. et al. (2025)	CPI in frozen dessert, 5–15%	CPI contained 84.69% protein; viscosity increased 2317 → 2709 cP (2.317 → 2.709 Pa·s) and melting time 47.78 → 51.05 min; 10% CPI gave highest overall acceptability	CPI affected matrix viscosity, melting behavior, and sensory response in an inclusion-dependent manner; these effects remain specific to the frozen-emulsion formulation
Chickpea – Nosworthy et al. (2020)	Extruded, cooked and baked chickpea	DIAAS-type scores: 82, 78 and 84; corresponding PDCAAS: 83.8, 75.2 and 80.0	Different processing treatments produced different protein-quality estimates, but DIAAS-type and PDCAAS remain methodologically distinct from true-ileal DIAAS
Microalgae – Benelhadj et al. (2016)	*Arthrospira platensis* protein isolate; pH 3–10	Nitrogen solubility 6.2 → 59.6 ± 0.7%; WAC 428.8 ± 15.4 g/100 g and OAC 252.7 g/100 g	Increasing net charge away from the near-isoelectric region was associated with greater protein dispersion and solubility; responses remain pH- and assay-dependent
Microalgae – Suarez Garcia et al. (2018)	*T. suecica* soluble protein extract	Protein extraction yield 22.5% at ∼0.6 kWh/kg (2.16 MJ/kg) dry biomass; extract contained 50.4% protein	Demonstrates energy-dependent recovery of a protein-rich soluble fraction, but co-extracted constituents prevent functionality from being assigned to protein alone
Microalgae – Le et al. (2025)	*N. oceanica* protein concentrate	Concentrate contained ∼86% crude protein (DW), with reduced lipid- and chlorophyll-associated fractions	Defatting and extraction enriched protein while removing lipid/pigment-associated components; improved functional or sensory performance cannot be inferred without direct testing
Microalgae – Nunes et al. (2020)	*T. chuii* in gluten-free bread	Bread volume decreased from 701 cm^3^ (control) to 612 cm^3^ at 2% inclusion; crumb b* changed from 19.2 to 27.1	Biomass incorporation produced simultaneous structural and color changes, demonstrating strong matrix dependence rather than a simple protein-enrichment effect
Microalgae –Qazi et al. (2021)	Microalgae-enriched wheat bread	DSEC IVPD 50.5–63.4% vs 68.5% control; literature-coefficient-derived DIAAS-type estimates 57–66 vs 46	Digestibility and calculated protein-quality responses depended on species, pretreatment, food matrix, and external digestibility coefficients; the estimates are not measured true-ileal DIAAS
Duckweed – Dhamaratana et al. (2025)	*W. globosa* biomass and protein fraction	Whole powder contained 23.93 ± 0.01% protein; recovered protein fraction contained 66.29 ± 1.42%	Fractionation substantially enriched protein relative to whole biomass, emphasizing the need to distinguish biomass composition from recovered protein ingredients
Duckweed – Duangjarus et al. (2022)	*W. globosa* protein/peptide extracts	Protein extract solubility reached 90.49% at pH 8; EC 71.29% at pH 4 and ES ∼98%	Functional responses were strongly condition-dependent; optimal solubility and emulsification occurred under different pH conditions
Duckweed – Nitiwuttithorn et al. (2024)	*W. arrhiza*; UAAE	Protein recovery 34.17 ± 0.32%; WHC 3.49 ± 0.34 g/g and OHC 3.26 ± 0.40 g/g	Extraction generated a functional protein-rich fraction, but no direct molecular mediator linking extraction to WHC/OHC was quantified
Duckweed – Seephua et al. (2025)	*W. globosa* in rice noodles	Protein 4.29 ± 0.30 → 8.39 ± 0.54% DW; operational IVPD 66.68 ± 0.36 → 81.51 ± 0.68%	Whole-biomass incorporation increased measured protein and operational IVPD, but the effects cannot be attributed specifically to isolated duckweed protein
Duckweed – Siriwat et al. (2023)	Protein hydrolysis with Alcalase	Solubility 57.07 ± 6.01 → 80.48 ± 6.24%; foaming 42.21 ± 1.12 → 55.00 ± 0.00%; OBC 4.65 ± 0.05 → 1.83 ± 0.01 g/g	Hydrolysis produced a clear functional trade-off: improved solubility/foaming at selected DH values accompanied lower oil-binding capacity at more extensive hydrolysis
Duckweed – Maag et al. (2025)	*L. gibba*; UAE + ultrafiltration	Maximum protein release 80.83%; protein yield 41.30% with 50.91% protein in the concentrate	Combined extraction and concentration improved protein recovery and enrichment, but these recovery metrics do not by themselves establish improved techno-functionality

**Note:**Table 1 presents representative quantitative primary evidence together with the principal comparative interpretation used in this review. The interpretation column distinguishes study-supported associations from intrinsic source effects; detailed analytical basis, assay conditions, protein-quality methodology, and food-matrix limitations are provided in Table 2, Table 3, Table 4. AEIP, alkaline extraction–isoelectric precipitation; CPI, chickpea protein isolate; DF, dry fractionation; DW, dry weight; EC, emulsifying capacity; ES, emulsifying stability; FC, foaming capacity; FS, foaming stability; HPH, high-pressure homogenization; IVPD, in vitro protein digestibility; OBC, oil-binding capacity; OHC, oil-holding capacity; SE, salt extraction; UAAE, ultrasound-assisted alkaline extraction; WAC, water-absorption capacity; WE, wet extraction. DH, degree of hydrolysis; DIAAS, digestible indispensable amino acid score; DSEC, SEC-based in vitro protein digestibility; IV-PDCAAS, in vitro protein digestibility-corrected amino acid score; LOX, lipoxygenase; OAC, oil-absorption capacity; PDCAAS, protein digestibility-corrected amino acid score; UAE, ultrasound-assisted extraction; WHC, water-holding capacity.

Table 1 summarizes representative primary studies that include measurable outcome data evaluating composition, processing, structure, digestibility, functionality or food-matrix behavior. Below, comparisons across sources are discussed with respect to material state, testing methodology, processing history and assay conditions, not source titles.

## 3 Chemical composition, protein fractions, and nutritional quality

Nutritional comparisons should be made on a clearly defined analytical basis. Protein concentration, amino acid composition, digestibility, and protein-quality values cannot be directly compared between any two ingredients without defining the form of ingredient, moisture basis, protein-determination method, nitrogen-to-protein conversion factor, prior-processing history, and digestion model used to calculate each value. Thus, rather than presenting the four protein sources like compositionally homogeneous ingredients, this review compares them on common analytical terms.

For lentil and chickpea, comparisons were made between whole seeds, flours, concentrates, isolates, and fractionated proteins. These ingredient forms differ in protein concentration, residual matrix constituents, and processing history, which can influence their physicochemical and techno-functional properties. Higa et al. (2024) found effects of pulse type and extraction route on protein yield, surface properties, functionality, in vitro protein digestibility (IVPD), and in vitro protein digestibility-corrected amino acid score (IV-PDCAAS). Ladjal-Ettoumi et al. (2016) found pH-dependent physicochemical and emulsifying properties among lentil and chickpea isolates. These data support an interpretation specific to ingredient form over assigning fixed nutritional/functionality to either crop.

Taxonomic resolution and analytical basis require particular attention when interpreting microalgal protein data. Species-level designations were therefore retained for experimental evidence, whereas “spirulina” was used only for commercial materials described using that term in the original source. Total‑nitrogen measurements include both protein nitrogen and non-protein nitrogen (NPN), including nucleic acids, pigments and inorganic nitrogen, and protein estimates obtained using a fixed nitrogen-to-protein conversion factor may therefore differ substantially from amino-acid-based protein measurements. In a primary experimental study of 12 marine microalgal species, Lourenço et al. (2004) quantified the distribution of intracellular nitrogen under different nitrogen regimes and growth phases and derived species- and condition-dependent N-to-protein factors ranging from 2.53 to 5.77. Combining the current experimental data with their previous dataset, Lourenço et al. (2004) reported an overall mean N-to-protein conversion factor of 4.78 ± 0.62 (*n* = 354) across species, treatments, and growth phases; accordingly, this value should not be interpreted as a universal factor for all microalgal biomass. Templeton and Laurens (2015) further demonstrated the effect of biomass composition and NPN on nitrogen-to-protein conversion. For *Chlorella vulgaris*, amino-acid-residue protein varied markedly across cultivation stages, from 34.59% dry weight (DW) in early-stage biomass to 10.36–10.62% in middle-stage technical duplicates and 8.45% in late-stage biomass, while the corresponding *k*_P_ values were 3.84 for early-stage biomass, 3.84 and 3.93 for the middle-stage technical duplicates, and 3.88 for late-stage biomass. These data demonstrate that protein estimates should be interpreted together with species, cultivation state, analytical method and the specific conversion factor used rather than by applying N × 6.25 or another group-average factor without qualification (Lourenço et al., 2004; Templeton & Laurens, 2015).

The same qualification applies to duckweed biomass. Appenroth et al. (2017) analyzed protein in freeze-dried biomass of six Lemnaceae species using Kjeldahl nitrogen and N × 6.25. The calculated crude protein content was around 20–35% DW for all species analyzed (Appenroth et al., 2017). Devlamynck et al. (2020) reported total nitrogen determined by Dumas and Kjeldahl nitrogen separately in *Lemna minor*. These samples were analyzed for crude protein (Kjeldahl N × 6.25) with values ranging from 15 ± 0% to 31 ± 1% DW depending on the cultivation medium used. Note that total nitrogen measured by Dumas was higher than Kjeldahl nitrogen because the analytical methods target different pools of nitrogen. Once again these findings illustrate that nitrogen-derived protein values must be interpreted alongside the nitrogen method and conversion factor used as well as cultivation condition and reporting basis (Appenroth et al., 2017; Devlamynck et al., 2020).

Amino-acid data should be qualified. Conventional acid hydrolysis does not quantitatively recover sulfur amino acids unless appropriate oxidation steps are taken, whereas tryptophan is analyzed using a separate procedure because it is unstable under acidic hydrolysis conditions. Allred and MacDonald (1988) performed oxidation with performic acid before acid hydrolysis for sulfur amino acids and used separate alkaline hydrolysis for tryptophan. Therefore, only when the analytical method was suitable for the amino acid of interest was the limiting-amino acid assignment deemed conclusive; otherwise, the limitation is noted in Table 2.

**Table 2:** Analytical basis, protein-quality evidence, and sources of interpretive bias for lentil, chickpea, microalgal, and duckweed proteins.

Panel A. Protein composition and analytical comparability				
Source/primary study	Material	Protein analytical basis	Representative result	Analytical implication/source of bias
Lentil – Nosworthy et al. (2018)	Red/green lentil flours; untreated and thermally processed	Dumas; N × 6.25; as-is	Crude protein: red lentil 25.13–26.86%; green lentil 23.93–25.67%	Values represent nitrogen-derived crude protein rather than amino-acid-residue protein; comparison with AA-based protein requires qualification
Chickpea – Nosworthy et al. (2020)	Untreated, extruded, cooked and baked chickpea flour	Dumas; N × 6.25; DM	Crude protein 19.93–22.10% DM across treatments	Fixed N × 6.25 assumes comparable protein-N contribution among treatments and does not separately quantify NPN
Microalgae – Taiti et al. (2024)	Dried *Arthrospira platensis* biomass	Kjeldahl; N × 6.25; DW	54.84 ± 0.13 g/100 g DW	NPN was not separately quantified; therefore, application of N × 6.25 may include nitrogen not incorporated into protein
Microalgae – Templeton and Laurens (2015)	*Chlorella vulgaris* biomass at different cultivation stages	Total N + amino-acid-residue protein; calculated *k*_P_; DW	AA-residue protein 34.59% early → 8.45% late; *k*_P_ 3.84–3.93	Biomass composition and cultivation stage alter the relationship between total nitrogen and amino-acid-residue protein, making a fixed conversion factor analytically unstable
Microalgae – Lourenço et al. (2004)	12 marine microalgal species	Total N + protein-bound AA; calculated N-to-protein factor	Factors 2.53–5.77; overall mean 4.78 ± 0.62	Species- and condition-dependent partitioning of nitrogen between protein and NPN prevents use of a universal microalgal conversion factor
Duckweed – Appenroth et al. (2017)	Freeze-dried biomass of six Lemnaceae species	Kjeldahl; N × 6.25; DW	Approximately 20–35% protein DW across species	Values are method- and conversion-factor-dependent; the same N × 6.25 factor may not represent equivalent AA-residue protein across species
Duckweed – Devlamynck et al. (2020)	*Lemna minor* across cultivation media	Dumas total N; Kjeldahl-N × 6.25; DW	Crude protein 15 ± 0 to 31 ± 1% DW	Dumas total N and Kjeldahl-N capture different nitrogen pools, demonstrating method-dependent apparent protein content
Duckweed – Dhamaratana et al. (2025)	*W. globosa* whole-biomass powder	Kjeldahl; N × 6.25; reported *w*/w	23.93 ± 0.01% protein; moisture 5.92 ± 0.04%	The value should be interpreted as Kjeldahl-derived crude protein of the specific whole-biomass material and should not be transferred directly to extracted fractions


Panel B. Amino-acid analytical basis and protein-quality evidence				
Source/primary study	Material	AA/digestibility basis	Representative result	Analytical implication/source of bias
Lentil – Nosworthy et al. (2018)	Extruded, cooked and baked red/green lentils	AA analysis with SAA/Trp-specific procedures; rat fecal protein digestibility	DIAAS-type: red 58/53/50; green 53/49/44	Fecal protein digestibility does not provide indispensable-AA-specific true ileal digestibility; values should therefore be described as DIAAS-type, not true-ileal DIAAS
Chickpea – Nosworthy et al. (2020)	Extruded, cooked and baked chickpea	AA analysis; true fecal protein digestibility	DIAAS-type: 82/78/84; PDCAAS 83.8/75.2/80.0	Fecal-protein-based scoring and PDCAAS use different digestibility concepts from true ileal DIAAS and should not be treated as interchangeable
Chickpea – Han et al. (2020)	Soaked/cooked chickpea	SAA oxidation + separate Trp; true ileal AA digestibility in growing pigs	DIAAS 67 for 6 months–3 years and 76 for older groups	Direct ileal indispensable-AA digestibility provides the amino-acid-specific physiological basis required for conventional DIAAS calculation
Lentil – Cargo-Froom et al. (2023)	Lentil at 40% in steam-pelleted pig diet	SID of individual AA in growing pigs	DIAAS 57.83; Trp limiting	Provides direct ileal AA evidence, but the steam-pelleted experimental diet is not equivalent to cooked lentil prepared as a human food
Microalgae – Qazi et al. (2021)	Microalgae-enriched breads	6 M HCl AA analysis; INFOGEST/SEC IVPD; literature digestibility coefficients	DSEC 50.5–63.4% vs 68.5% control; calculated DIAAS-type 57–66 vs 46	External digestibility coefficients and cross-species extrapolation prevent direct equivalence with measured ileal DIAAS; AA preparation also limits definitive interpretation of some limiting AAs
Duckweed – Seephua et al. (2025)	Rice noodles with 1–5% *W. globosa*	Operational INFOGEST IVPD; no AA profile supporting standardized scoring	IVPD 66.68 ± 0.36 → 81.51 ± 0.68%	IVPD indicates operational digestive release under the applied model but cannot be interpreted as PDCAAS, DIAAS, or amino-acid bioavailability

**Note:** Protein values retain the analytical method, nitrogen-to-protein conversion factor, and reporting basis of the original studies. Nitrogen-derived crude protein and amino-acid-residue protein are analytically distinct, and study-specific conversion factors should not be transferred across species. DIAAS-type denotes scores calculated using fecal protein digestibility or literature-derived coefficients, whereas DIAAS refers to scores based on ileal indispensable-amino-acid digestibility. DW, dry weight; DM, dry matter; NPN, non-protein nitrogen; SAA, sulfur amino acids; IVPD, in vitro protein digestibility; PDCAAS, protein digestibility-corrected amino acid score; DIAAS, digestible indispensable amino acid score. AA, amino acid; DSEC, SEC-based in vitro protein digestibility; INFOGEST, standardized static gastrointestinal digestion protocol; SEC, size-exclusion chromatography; SID, standardized ileal digestibility.

Digestibility values were also maintained separately. Again, IVPD is just an operational estimate made in vitro and is not a protein-quality score in itself. INFOGEST 2.0 standardizes the static in vitro digestion methodology to mimic gastrointestinal digestion, and INFOGEST Quant applies the INFOGEST digestion methodology further to total protein digestibility, individual amino-acid digestibility and in vitro DIAAS when amino acids are quantified correctly. PDCAAS merges amino acid scoring with overall protein digestibility, while Food and Agriculture Organization of the United Nations (FAO) DIAAS is calculated from the digestibility of individual indispensable amino acids using true ileal values where possible. Results from these assays are not directly comparable (Brodkorb et al., 2019; Egger et al., 2026; FAO, 2013).

For lentil and chickpea, protein-quality evidence should be interpreted according to the digestibility basis used in each study. Nosworthy et al. (2018) reported DIAAS-type scores based on true fecal protein digestibility of 58, 53, and 50 for extruded, cooked, and baked red lentil flour, respectively, and 53, 49, and 44 for the corresponding green lentil flours. Using the same fecal-protein-based approach, Nosworthy et al. (2020) reported scores of 82, 78, and 84 for extruded, cooked, and baked chickpea flour, respectively. Although the original studies referred to these values as DIAAS, they were not calculated using directly measured true ileal digestibility of individual indispensable amino acids. They should therefore be distinguished from ileal DIAAS as defined by FAO (2013). A targeted search for ileal DIAAS evidence in cooked lentil and chickpea identified direct evidence for cooked chickpea but not for cooked lentil prepared as a human food. Han et al. (2020) measured true ileal digestibility of individual amino acids in cooked chickpea using T-cannulated growing pigs and reported DIAAS values of 67 for children aged 6 months–3 years and 76 for older children, adolescents, and adults. No directly comparable true-ileal DIAAS study for cooked lentil prepared for human consumption was identified in the targeted search. Cargo-Froom et al. (2023) reported a DIAAS of 57.83 for Canadian lentil based on standardized ileal amino-acid digestibility measured in growing pigs; however, the lentil was incorporated at 40% into a steam-pelleted experimental diet processed at 80 °C rather than prepared as a cooked human food. This value was therefore retained as indirect ileal-digestibility evidence and should not be interpreted as validation of DIAAS for cooked lentil foods. Neither of these ileal values is directly comparable with the fecal-protein-based DIAAS-type scores reported by Nosworthy et al., 2018, Nosworthy et al., 2020. At the fraction level, chickpea crude protein, albumin, globulin, and glutelin differ in structure and functionality (Gao et al., 2025; Soto-Madrid et al., 2023), as well as in their responses after simulated gastrointestinal digestion (Gao et al., 2025). However, simulated post-digestion responses should not be interpreted as evidence of intestinal absorption or standardized protein quality when IVPD, PDCAAS, DIAAS, or amino-acid bioavailability were not measured.

Antinutritional factors provide additional context for pulse-protein quality, but causal attribution requires measurement in the same material. Trypsin inhibitors, phytate, tannins, and related constituents may affect enzyme activity or nutrient accessibility. However, because Nosworthy et al., 2018, Nosworthy et al., 2020 did not quantify these factors in the samples used for their protein-quality measurements, changes in the study-reported fecal-protein-based DIAAS-type scores should not be attributed directly to antinutritional-factor reduction. Processing effects should instead be interpreted as the combined result of protein structural changes, matrix interactions, and any measured changes in antinutritional components.

Digestibility data for microalgae have been mostly limited to protein accessibility, extraction-dependent release, and in vitro digestion instead of established protein-quality assessments. Robust cell walls may impede enzyme access to proteins in microalgae like *Chlorella* and *Nannochloropsis*. Cell rupture and protein extraction methods improve protein release; however, these methods can modify pigments, lipids, and protein conformation. Le et al. (2025) found that acetone defatting followed by alkaline extraction at pH 12.0 and acid precipitation at pH 2.0 yielded a *Nannochloropsis oceanica* protein concentrate that had about 86% crude protein on a dry-weight basis. Lipid content and chlorophyll-associated pigments were also decreased by this treatment, creating a less-green, yellowish powder (Le et al., 2025). Changes in composition and color could factor into future food application prospects, though these results did not assess consumer sensory acceptance. The above example demonstrates how protein recovery is tied to the removal of lipid- and pigment-associated cellular material, while sensory impacts need to be assessed independently. For this reason, data found in literature for microalgae are designated as IVPD, INFOGEST/static digestion, protein accessibility, or extraction-dependent release if digestible indispensable amino acids have not been measured using an appropriate protein-quality matrix (Egger et al., 2026).

Cell-wall destruction is an essential preprocessing step to improve microalgal protein accessibility. Bead milling, high-pressure homogenization, and ultrasonication are mechanical methods of cell disruption by physical forces. Pulsed electric fields and enzymatic hydrolysis treatments can make cell membranes more permeable under milder conditions. Further alkaline extraction or defatting-extraction treatments can enhance protein release while simultaneously reducing co-extracted lipid or pigments. Degree of disruption should be optimized because inadequate treatments restrict enzyme access, whereas over-processing may promote aggregation, oxidation, co-extraction of pigments, or loss of functional quality (Ali et al., 2025; Le et al., 2025; Prates, 2025). Duckweed composition should likewise be interpreted at the species, cultivation-system, and ingredient-form levels. Controlled aquatic or hydroponic cultivation intended for human consumption is different than wastewater-remediation-type systems because water quality, growth medium, nitrate and oxalate content, contaminants, microbial quality, and ingredient traceability affect food safety and thus suitability (Appenroth et al., 2017; Devlamynck et al., 2020; EFSA NDA Panel, 2021; EFSA NDA Panel, 2022). Protein extraction and recovery also cannot be predicted from biomass protein concentration alone. Protein extraction of cultured *Wolffia arrhiza* using ultrasound at pH 8.5 (duckweed to solution ratio of 1:6) resulted in 16.16 ± 0.21% production yield, 10.28 ± 0.06% protein yield, 34.17 ± 0.32% protein recovery, and produced an extract that contained ∼65% protein (Nitiwuttithorn et al., 2024). Ultrasonic extraction of protein from *Wolffia globosa* at 30 mL/g, 25 min, 78% amplitude had a protein extraction yield ∼2.17 times greater than alkali extraction only (Siriwat et al., 2023). Examples like these are why extraction conditions should be reported with values for composition and functionality.

RuBisCO contributes to the photosynthetic protein fraction of duckweed but should not be considered universally dominant: RuBisCO varies greatly in relative abundance between species, cultivation conditions, tissue makeup, harvest time-point, and protein-extraction methods (Maag et al., 2025; Muller et al., 2025). Supporting digestibility evidence for duckweed was also less standardized compared to lentil and chickpea. For example, fractions of *Wolffia globosa* and enzyme-treated *Lemna minor* provided simulated digestion and enzymatic-accessibility data but this review did not identify food-grade duckweed protein ingredients with reported standardized ileal DIAAS values among the reviewed primary studies (Dhamaratana et al., 2025; Egger et al., 2026; Pauliukaitytė et al., 2026).

Table 2 maintains reported material form, protein-determination basis, conversion factor used to calculate nitrogen-to-protein, reporting basis, and digestibility metric while also calling out the main qualification to take into consideration when comparing these findings between studies. Keep in mind that these four protein sources should not be implicitly ranked based on their intrinsic nutrition until these analytical and processing variables are aligned.

## 4 Processing-induced structural modifications and structure-function relationships

Processing changes protein conformation, aggregation, particle size, molecular-weight distribution, surface hydrophobicity, charge, thermal properties, residual non-protein constituents, and ultimately solubility, emulsification, foaming, gelation, water/oil retention, viscosity, and colloidal stability. The latter are also assay-dependent and performance can be significantly altered by pH, ionic strength, concentration, hydration, homogenization, and other conditions of the applied test. Structural evidence is therefore understood relative to ingredient form, processing history, and assay conditions instead of as an intrinsic property of the source material (Ma et al., 2022; Nikbakht Nasrabadi et al., 2021).

Lentil protein particle properties and dispersion performance can be modified by mechanical treatment in defined processing conditions. Malterre et al. (2024) studied red lentil protein isolate dispersions formed by in-line, high-shear rotor–stator mixing at 10,200 rpm for up to 15 min. Particle diameter decreased from 5.13 ± 0.58 to 1.72 ± 0.01 μm while solubility increased from 46.87 ± 1.55% to 68.42 ± 8.04%. Surface hydrophobicity increased with shear mixing, and separation rate decreased from 71.23 ± 18.15 to 24.16 ± 2.58% h^−1^. Malterre et al. (2025) applied single-pass high-pressure homogenization to lentil protein isolate suspensions. Treatment at 180 MPa decreased particle diameter from 10.7 ± 1.1 to 0.33 ± 0.06 μm, while protein solubility increased from 62.5 ± 2.63% for the untreated control to 95.3 ± 0.68%. The observed increase in surface hydrophobicity accompanied particle-size reduction and improved dispersion properties. However, solubility did not increase monotonically with pressure and was highest (96.4 ± 0.55%) at 150 MPa. These studies show relationships between particle disruption, surface properties, and functional performance that are dependent on processing rather than universal across lentil protein ingredients.

Fractionation of chickpea offers another example where protein functionality has been shown to rely on properties of the recovered fractions, rather than identity of protein source alone. For example, Gao et al. (2025) compared crude protein (CP), albumin, globulin, and glutelin obtained by Osborne-type fractionation methods. Reported total sulfhydryl contents were 16.62 ± 1.09, 33.81 ± 0.60, 14.40 ± 2.16, and 26.51 ± 0.49 μmol/g of each fraction, respectively. Protein solubility at pH 3 was determined to be 8.26 ± 0.95% for CP, 10.55 ± 1.40% for albumin, 4.45 ± 0.70% for globulin, and 8.26 ± 0.70% for glutelin. Using the foaming assay reported by Gao et al. (2025) at pH 7, albumin had a foaming capacity of 70 ± 3% and stability of 41 ± 1%. Using the emulsification assay at pH 7, CP, glutelin, albumin, and globulin had emulsifying-capacity values of 18.27 ± 0.57, 15.12 ± 0.51, 12.62 ± 0.62, and 9.95 ± 0.49, respectively; glutelin had the greatest emulsion-stability response. Each assay was conducted using defined protein concentrations, pH, oil fraction, and homogenization parameters. Values reported here therefore should not be compared directly to values reported in other studies or generalized across assays. Fractionation also simultaneously altered composition as well as many structural characteristics of the material, making it impossible to attribute any differences observed to changes in any single structural variable (Gao et al., 2025).

Commercial protein ingredients show similar complexities when trying to attribute functional differences to a single processing factor. De Angelis et al. (2024) reported pooled final storage moduli (*G*′) of 2183 ± 986 Pa for dry-fractionated (DF) ingredients and 768 ± 1340 Pa for wet-extracted (WE) ingredients. Although a statistical difference between the pooled means was reported by De Angelis et al. (2024), individual values from each group overlapped greatly, with *G*′ ranging from 0.11 to 3563 Pa among WE ingredients and from 974 to 4390 Pa among DF ingredients. Further, botanical species, suppliers, protein content, and residual matrix composition were not equivalent between groups. The pooled comparison therefore neither establishes a consistent performance advantage due to dry fractionation nor should be used to draw conclusions about the performance of dry-fractionated versus wet-extracted ingredients outside of this specific commercial set (De Angelis et al., 2024).

Functional behavior of microalgal proteins appears to be largely dependent on the species being investigated, the form of ingredient produced, and assay conditions. For example, Benelhadj et al. (2016) found that nitrogen solubility of *Arthrospira platensis* protein isolate increased with increasing pH from 6.2% at pH 3 to 59.6 ± 0.7% at pH 10. pH-dependent changes in ζ-potential and nitrogen solubility agreed with changes expected from electrostatic impacts on protein dispersion. Reported water absorption capacity, oil absorption capacity, and minimum gelation concentration values for this isolate were 428.8 ± 15.4 g/100 g at pH 10, 252.7 g/100 g, and 12% (w/w), respectively. These endpoints were generated using dissimilar assay conditions, and so cannot be directly compared as metrics of the same functional mechanism.

Soluble extracts containing proteins recovered from *Tetraselmis suecica* have been described by Suarez Garcia et al. (2018). Their method employed controlled bead milling at ∼25 °C, pH 6.5, which achieved protein extraction yields of 22.5% with a specific energy consumption of ∼0.6 kWh/kg (∼2.16 MJ/kg) dry biomass. This extract contained 50.4% protein and exhibited interfacial activity and thermal-gelation behavior under the assays described. Many carbohydrates and other matrix components were also extracted in this process, so the observed functionality cannot be entirely attributed to protein components. Contrasting this work, Le et al. (2025) enriched proteins from *Nannochloropsis oceanica* to approximately 86% crude protein on a dry basis by employing acetone defatting, alkaline extraction, and acid precipitation. While this study supports extraction-dependent protein enrichment and pigment reduction, it did not independently verify improved techno-functional or sensory metrics.

Properties of duckweed protein can vary dependent upon conditions of recovery as well as any additional modification thereafter. Nitiwuttithorn et al. (2024) reported that ultrasound-assisted alkaline extraction of *Wolffia arrhiza* at pH 8.5 with a duckweed-to-solution ratio of 1:6 resulted in 34.17 ± 0.32% protein recovery. The UAAE_1/6 extract exhibited a water-holding capacity of 3.49 ± 0.34 g/g at pH 7 and an oil-holding capacity of 3.26 ± 0.40 g/g at pH 4.5. Emulsifying capacity and stability at pH 7 were measured at 100.00 ± 0.00% and 91.53 ± 0.87%, respectively. These results highlight that extraction route should also be reported alongside sample identity and pH at assay when reporting functionality of duckweed protein.

Modification of *Wolffia globosa* protein after recovery via enzymatic hydrolysis with Alcalase and Protamex was investigated by Siriwat et al. (2023). Hydrolysis was shown to modify the molecular-weight distribution and Fourier-transform infrared (FTIR)-derived secondary structural characteristics of the isolate. Modifications to functionality include an increase in solubility at pH 7 from 57.07 ± 6.01% for nonhydrolyzed concentrate to 80.48 ± 6.24% at degree of hydrolysis (DH) 9% after Alcalase treatment. Foaming activity was greatest at DH 3%, increasing from 42.21 ± 1.12% to 55.00 ± 0.00%. Oil-binding capacity was reduced after Alcalase treatment (4.65 ± 0.05 to 1.83 ± 0.01 g/g, DH 9%). Similar reductions in emulsifying stability were observed with increasing DH. Taken together, these findings demonstrate how enzymatic modification can improve certain functional endpoints while diminishing others, and that the proper method of treatment will be dependent on desired application within a food-matrix rather than attempting to maximize hydrolysis.

Therefore, the evidence-base for post-extraction functional modification is uneven across the four protein-source categories. Encompassed within the studies reviewed here, lentil and chickpea have been subjected to more varied mechanical, enzymatic, thermal, fermentation, and extrusion-related modification strategies while the primary studies on microalgae that were retained for this review focus primarily on cell disruption, fractionation, defatting, protein recovery, and enrichment (versus modification of an already recovered protein ingredient) (Le et al., 2025; Malterre et al., 2024, Malterre et al., 2025; Suarez Garcia et al., 2018). Duckweed provided some evidence of post-extraction modification (via controlled enzymatic hydrolysis of *Wolffia globosa* protein) (Siriwat et al., 2023), but glycosylation, phosphorylation, covalent conjugation, and other defined post-extraction physical modification strategies were not represented among the primary food-protein studies retained for this review. This asymmetry should be regarded less as evidence that such approaches would be ineffective for microalgal or duckweed proteins, and more as a major gap in the literature. Future work should evaluate well-characterized concentrates/isolates using matched, untreated controls along with quantitative structural measurements and standardized functional assays to understand if/how post-extraction modification leads to reproducible structure–function improvements in these aquatic systems.

Table 3 summarizes selected quantitative processing-structure-function evidence and associated assay conditions required for interpretation and comparison among studies. Improvements in these selected properties have been linked to processing-induced structural changes; however, this response is often condition dependent and indicative of trade-offs between solubility/emulsification/foaming/oil retention/gelation capacity. Functional changes should therefore not be linked to an individual structural mediator without sufficient evidence to isolate said mechanism. Fig. 1 provides a quantitative visualization of three select study-level processing-structure-function relationships reported in Table 3. Rather than report a generalized mechanistic outcome, the figure instead plots the measured response of lentil, microalgal, and duckweed protein systems treated with high pressure homogenization, pH variation, or controlled enzymatic hydrolysis, respectively. Reported results are improvements or functional trade-offs that were observed under published experimental conditions. Panels should not be interpreted as pooled cross-source comparisons as the protein source, treatment, and assay conditions differ between studies.

**Table 3:** Quantitative processing-structure-function evidence, assay conditions, and mechanism-oriented interpretation.

Source/study	Material/processing	Functional assay conditions	Measured structural/physicochemical change	Functional consequence and mechanistic interpretation
Lentil – Malterre et al. (2025)	LPI (79.63% protein), 5% suspension; single-pass two-stage HPH, 0–180 MPa, pH 6.8	Hydrophobicity: ANS, 0.01 M phosphate, pH 7; stability by analytical centrifugation; oil fraction NA; ionic strength NR	Particle diameter 10.7 ± 1.1 → 0.33 ± 0.06 μm; surface hydrophobicity 614 ± 66 → 1312 ± 82	Solubility increased 62.5 ± 2.63 → 95.3 ± 0.68% and separation rate decreased 8.55 → 4.92% h^−1^. Particle disruption and increased surface exposure were associated with improved dispersion/solubility, but solubility peaked at 150 MPa, demonstrating a non-monotonic response. Individual structural mediators were not independently isolated
Chickpea – Gao et al. (2025)	CP, albumin, globulin and glutelin; Osborne fractionation	Solubility 1 mg/mL, pH 1–11; foaming 10 mg/mL, 8000 rpm, 3 min; emulsification 10 mg/mL, protein:soybean oil = 6:2 mL, 8000 rpm, 3 min; ionic strength NR	Total -SH differed among fractions, ranging from 14.40 to 33.81 μmol/g; fractionation generated compositionally and structurally distinct protein fractions	Solubility at pH 3 ranged 4.45–10.55%; albumin FC/FS at pH 7 = 70 ± 3/41 ± 1%; EC = 9.95–18.27 mL/g and ES = 48.55–67.14 min. Fraction-specific molecular composition and interaction potential are consistent with differing functionality, but fractionation altered several variables simultaneously and no single mediator can be assigned as causal
Mixed plant proteins – De Angelis et al. (2024)	Commercial DF (*n* = 9) vs WE (*n* = 8) ingredients	Solubility 5% *w*/*v*, pH 3/5/7/9; foaming 5%, 24,000 rpm, 90 s, pH NR; emulsion 1% protein, 24,000 rpm, 3 min, oil added to phase inversion, oil identity NR; rheology 15% w/v, 0.5% strain; ionic strength NR	No single structural mediator was isolated; DF and WE groups simultaneously differed in botanical origin, supplier, protein content, residual matrix constituents, and processing history	Final *G*′ was 2183 ± 986 vs 768 ± 1340 Pa, but individual ranges overlapped strongly (DF 974–4390; WE 0.11–3563 Pa). Apparent rheological differences therefore reflect a composite ingredient effect and cannot be attributed mechanistically to fractionation route alone
Microalgae – Benelhadj et al. (2016)	*A. platensis* protein isolate; pH-dependent characterization	ζ-potential at 0.5% isolate; emulsification: 5 g isolate/50 mL buffer +50 mL sunflower oil, 22,000 rpm, 2 min; gelation 2–20% *w*/w; ionic strength NR	ζ-potential was near zero around pH 3 and became more negative at alkaline pH	Nitrogen solubility increased 6.2 → 59.6 ± 0.7% from pH 3 to 10; WAC = 428.8 ± 15.4 g/100 g and OAC = 252.7 g/100 g. Greater electrostatic repulsion away from the near-isoelectric region is consistent with reduced protein–protein association and improved dispersion, although functional endpoints remain assay-dependent
Duckweed – Nitiwuttithorn et al. (2024)	*W. arrhiza* UAAE at pH 8.5, 1:6; protein recovery 34.17 ± 0.32%	WHC pH 7; OHC pH 4.5; emulsification: 1% extract, pH 7/8.5, sunflower oil:aqueous = 2:1 (*v*/v); Ultra-Turrax T25, 1 min + 2 min after second oil addition; speed NR; ionic strength NR	No direct molecular structural mediator was quantified in the tested extract	WHC = 3.49 ± 0.34 g/g, OHC = 3.26 ± 0.40 g/g, EC = 100.00 ± 0.00%, ES = 91.53 ± 0.87%. The data establish extraction- and assay-condition-specific functionality but do not support attribution to a defined molecular mechanism
Duckweed – Siriwat et al. (2023)	*W. globosa* concentrate; Alcalase/Protamex hydrolysis, DH 3–9%	Solubility 1% *w*/*v*, pH 3–11; foaming 1%, 10,000 rpm, 1 min; emulsification 5% protein + soybean oil 1:1, 10,000 rpm, 1 min; ionic strength NR	Hydrolysis changed molecular-weight distribution and FTIR-derived secondary structure; α-helix 26.55 → 40.26% and random coil 47.06 → 36.03%	Solubility increased 57.07 ± 6.01 → 80.48 ± 6.24%; foaming increased 42.21 ± 1.12 → 55.00 ± 0.00% at selected DH, whereas OBC decreased 4.65 ± 0.05 → 1.83 ± 0.01 g/g at DH 9%. Concurrent structural and functional changes demonstrate a DH-dependent trade-off, but direct causal mediation by any single structural variable was not independently tested

**Note:** Assay conditions are retained because pH, ionic strength, protein concentration, oil fraction, and homogenization technique can significantly influence functionality assays. NR = not reported, NA = not applicable. Structural indicators were included only if they were measured directly. Mechanistic statements denote experimentally supported associations or physicochemically consistent interpretations and should not be construed as proof that a particular structural mediator independently caused the observed functional response. ANS = 8-anilino-1-naphthalenesulfonic acid; CP = crude protein; DF = dry fractionation; DH = degree of hydrolysis; EC = emulsifying capacity; ES = emulsifying stability; FC = foaming capacity; FS = foaming stability; HPH = high-pressure homogenization; LPI = lentil protein isolate; OBC = oil-binding capacity; OHC = oil-holding capacity; WAC/WHC = water absorption/water holding capacity; WE = wet extraction. FTIR = Fourier-transform infrared spectroscopy; OAC = oil-absorption capacity; –SH = sulfhydryl group; UAAE = ultrasound-assisted alkaline extraction.

**Fig. 1:**
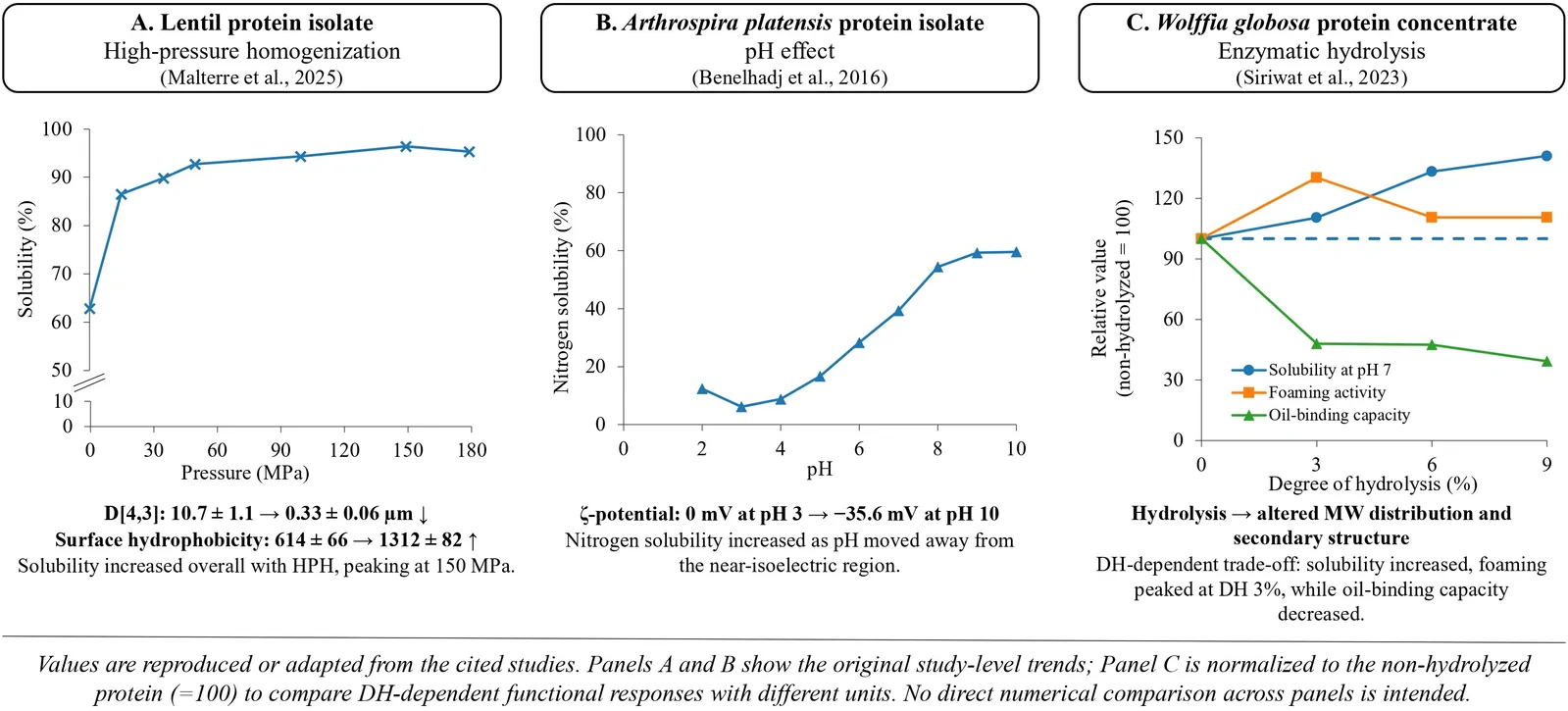
Quantitative study-level trends in processing-structure-function responses of alternative protein ingredients. (A) High-pressure homogenization of lentil protein isolate increased solubility overall, with the maximum response observed at 150 MPa, while particle diameter decreased from 10.7 ± 1.1 to 0.33 ± 0.06 μm and surface hydrophobicity increased from 614 ± 66 to 1312 ± 82 (Malterre et al., 2025). The y-axis in Panel A is broken between 10 and 50% to improve visualization of the treatment-dependent response while retaining the zero baseline. (B) Nitrogen solubility of *Arthrospira platensis* protein isolate increased as pH moved away from the near-isoelectric region, while ζ-potential changed from approximately 0 mV at pH 3 to −35.6 mV at pH 10 (Benelhadj et al., 2016). Intermediate nitrogen-solubility values in Panel B were digitized from the published Figure 3, whereas directly reported values were retained where available. (C) Enzymatic hydrolysis of *Wolffia globosa* protein produced degree-of-hydrolysis-dependent functional trade-offs, with increased solubility, maximum foaming response at DH 3%, and progressively lower oil-binding capacity (Siriwat et al., 2023). Functional responses in Panel C were normalized to the corresponding non-hydrolyzed protein (=100) because the original endpoints were reported in different units. Values are reproduced, digitized, or normalized from the cited studies as indicated and should be interpreted as study-specific trends rather than direct cross-source numerical comparisons. Concurrent structural and functional changes are presented as study-level associations and do not establish independent causal mediation.

Fractionation route is one such decision. Dry fractionation requires little to no water or solvent and will generally leave a less extensively modified protein state behind, but generates protein-rich flours/concentrates with more starch, fiber, lipid, pigment, and other non-protein solids. Wet extraction permits higher enrichment and more standardized starting formulations but requires more water, pH adjustment, washing or neutralization, separation, and drying, and severe conditions may promote denaturation or protein aggregation. Differences in protein structure and techno-functional behavior were reported between commercial dry-fractionated and wet-extracted ingredients by De Angelis et al. (2024). In this comparison, however, flours originated from different botanical sources, were provided by different suppliers, and had different protein purity and residual constituent contents. Thus, observed differences cannot be attributed solely to the fractionation route. Dry-wet hybrid processes can limit the amount of material exposed to wet conditions but will still need to be optimized according to their application. Fractionation should be selected based on desired purity/recovery, resource use, structural preservation, and target-matrix performance in addition to protein yield (De Angelis et al., 2024; Dumoulin et al., 2021; Schutyser et al., 2025).

Broader still, numerous post-extraction modification strategies discussed throughout this protein section encompass enzymatic hydrolysis, fermentation, heat treatment, and extrusion (Sha & Xiong, 2020), among others. All of these processes can alter molecular size, conformation/aggregation, and intermolecular interactions to enhance some desired properties while reducing others, especially under more extreme conditions (Gasparre et al., 2025; Hülsebusch et al., 2025; Yousefi et al., 2025; Zang et al., 2025). Again, these are not uniformly utilized across all four protein groups discussed in this review and will not be expected to elicit uniform responses when applied to microalgal or duckweed proteins. Individual studies will have to verify this. Major mechanisms, pros, and cons of representative processing/modification approaches are summarized in Supplementary Table S1. On a final note, processing should be designed with intended structural and functional specifications of the food matrix in mind, not just protein recovery.

## 5 Food-matrix performance and application-oriented protein design

Application performance is dictated not by source identity but rather by how well a defined protein ingredient meets the technological needs of the target matrix. For this reason, studies describing matrix-level proof-of-concept are prioritized over those extrapolating from benchtop functional assays, and Table 4 includes only studies wherein a defined protein ingredient or protein-rich biomass was directly evaluated in a real food system.

**Table 4:** Food-matrix evidence, matrix-level interpretation, and validation needs for selected alternative protein ingredients.

Source/primary study	Ingredient and food matrix	Incorporation/processing condition	Quantitative or directly observed response	Matrix-level interpretation/mechanistic basis	Remaining validation need
Lentil – Caldeira et al. (2024)	Wet-processed *Lens culinaris* protein concentrate; fish-like plant-based croquette	Alkaline extraction/acid precipitation; optimized concentrate subsequently evaluated in a croquette	Dried concentrate contained 85% protein DW at 14% mass yield; first scale-up reproduced the process. Sensory acceptance, flavor and texture were similar to the commercial clean-taste concentrate	Directly demonstrates compatibility of the recovered lentil concentrate with a structured food matrix. The acceptable texture/sensory response reflects matrix-level performance, but the contribution of individual molecular or interfacial mechanisms was not isolated	One concentrate and one croquette system; sensory and processing performance should not be generalized to other meat analogues
Chickpea – Arshad P. et al. (2025)	Chickpea protein isolate in oat-drink-based frozen dessert	CPI at 5, 10 and 15%	CPI: 84.69% protein, 60.2% solubility, 62.37% FC and 69.54% EC; viscosity increased 2317 → 2709 cP, overrun decreased 31.56 → 28.78%, melting time increased 47.78 → 51.05 min; 10% CPI gave highest overall acceptability	Higher CPI inclusion was associated with greater continuous-phase viscosity and longer melting time, consistent with increased matrix structuring and water immobilization. The study does not isolate whether protein hydration, interfacial effects, or interactions with the stabilizer system were dominant	Responses depend on oat-drink composition, stabilizer system, CPI preparation, and inclusion level
Microalgae – Nunes et al. (2020)	*Tetraselmis chuii* biomass in gluten-free bread	0, 1, 2 and 4% *w*/w	Bread volume changed from 701 cm^3^ control to 642, 612 and 640 cm^3^; crumb b* changed from 19.2 to 16.2, 27.1 and 22.2, respectively	Biomass incorporation altered loaf structure and color. The volume response is consistent with matrix interference/dilution effects, whereas color changes reflect biomass-associated constituents; specific starch-protein or gas-cell mechanisms were not directly isolated	Sensory acceptability and EU-authorized conditions of use must be considered separately from experimental feasibility
Microalgae – Qazi et al. (2021)	Cell-wall-disrupted *M. gaditana*, *T. chuii* and *C. vulgaris* biomass in wheat bread	Raw or ethanol-treated biomass; 12% wheat-flour substitution	Bread DSEC IVPD 68.5 ± 0.6% control vs 50.5–63.4% microalgae breads. Protein content increased ∼11–36%; calculated DIAAS-type values 57–66 vs 46 control	Species and pretreatment altered the combined matrix response. Cell disruption and biomass composition may affect digestive accessibility, but the calculated DIAAS-type values depend on external coefficients and do not establish a directly measured digestive mechanism in the breads	DSEC IVPD and calculated DIAAS are not equivalent to measured true-ileal DIAAS; 12% incorporation may not represent commercially optimal sensory levels
Duckweed – Seephua et al. (2025)	*W. globosa* whole-biomass powder in rice noodles	Watermeal replaced tapioca starch at 1, 3 and 5 percentage points of total formulation; 62.5% added water; WF5 ≈ 13.3% of nominal non-water ingredient blend before drying	Protein DW: 4.29 ± 0.30% control → 8.39 ± 0.54% WF5 (∼1.96-fold); operational IVPD 66.68 ± 0.36 → 81.51 ± 0.68%	Whole-biomass inclusion produced inclusion-level-dependent increases in measured protein and operational IVPD, but the response cannot be assigned specifically to duckweed protein because biomass composition and exact protein mass balance were not independently resolved	Protein content of the specific powder batch was not reported; exact protein mass balance cannot be reconstructed. Whole-biomass results should not be extrapolated to purified duckweed protein

**Note:**Table 4 includes only studies in which a defined lentil, chickpea, microalgal, or duckweed ingredient was evaluated in an actual food matrix. Extraction- or modification-only studies are summarized in 3, 4 and Table 3 and are not treated as direct evidence of food-product performance. Quantitative results should not be compared numerically across rows because ingredient form, incorporation level, matrix composition, processing conditions, analytical endpoint, and sensory methodology differ among studies. CPI, chickpea protein isolate; DIAAS, digestible indispensable amino acid score; DSEC, SEC-based in vitro protein digestibility; DW, dry weight; EC, emulsifying capacity; FC, foaming capacity; IVPD, in vitro protein digestibility; WF5, formulation containing 5 percentage points of watermeal powder.

A handful of primary food-matrix studies are highlighted below to demonstrate how ingredient performance can differ with changes in formulation and processing parameters. Caldeira et al. (2024) produced a lentil protein concentrate (85% protein on a dry basis) with a mass yield of 14% and evaluated it as an ingredient in a fish-analogue plant-based croquette. Similar sensory scores were reported for acceptance, flavor and texture compared with those achieved with a commercial clean-taste protein concentrate. Arshad P. et al. (2025) evaluated chickpea protein isolate added to an oat-based frozen dessert and noted formulation-dependent differences in physical and sensory properties. Nunes et al. (2020) investigated the incorporation of *Tetraselmis chuii* into gluten-free bread and noted alterations to bread volume and crumb color. In a separate study, Qazi et al. (2021) replaced 12% wheat flour with cell-wall-disrupted microalgal biomass and observed size exclusion chromatography (SEC)-based in vitro protein digestibility (DSEC) values ranging from 50.5 to 63.4% for the supplemented breads, compared to 68.5% for the unsupplemented control.

Qazi et al. (2021) also reported literature-coefficient-derived DIAAS-type estimates of 57–66 for the microalgae-enriched breads and 46 for the control. These estimates were not based on true ileal digestibility of individual indispensable amino acids measured directly in the tested breads. Instead, published digestibility coefficients were applied: wheat-based coefficients were derived from pig data, whereas the microalgal component was estimated using digestibility data reported for *Spirulina platensis* in humans. Because these coefficients were extrapolated to breads containing *Tetraselmis chui*, *Chlorella vulgaris*, or *Microchloropsis gaditana*, the resulting scores include cross-species extrapolation and should be interpreted as calculated DIAAS-type estimates rather than directly measured ileal DIAAS (Qazi et al., 2021). In addition, the reported amino-acid preparation used conventional acid hydrolysis and did not describe separate oxidation for sulfur amino acids or a tryptophan-specific hydrolysis procedure, which further limits definitive interpretation of the limiting amino acid.

Watermeal powder was used instead of tapioca starch at 1, 3, and 5 percentage points of a formulation with 62.5% added water in rice-noodle experiments by Seephua et al. (2025). Therefore, WF5 translated to approximately 13.3% of the nominal non-water ingredients mixture prior to drying - not 5% of dry ingredients. The authors also reported proximate composition on a dry-weight basis. Protein was 4.29 ± 0.30% in the control and 8.39 ± 0.54% in WF5, approximately a 1.96-fold increase. Operational in vitro protein digestibility increased from 66.68 ± 0.36% to 81.51 ± 0.68%. Accounting for both the wet-formulation basis used in the study and the dry-analysis basis, the reported near-doubling of protein should not be misinterpreted as resulting from a 5% dry-weight inclusion. Protein content of the *W. globosa* powder used was not reported; therefore, an accurate protein mass balance cannot be reconstructed independently (Seephua et al., 2025). Each publication described above exemplifies how protein enrichment, digestibility, structure/color, and sensory characteristics can be affected differently by source, level of incorporation, pretreatment techniques, and protein-addition matrix. Selection of ingredients based on some of these attributes, as proposed above, also seems to depend on requirements unique to a target-vehicle. Structured/meat analog products need water/oil-holding capacity, some binding ability for cohesiveness, and appropriate firmness; beverage and frozen-emulsion systems rely on solubility, dispersion stability, favorable viscosity contribution, flavor neutrality, and minimal sedimentation; starch-heavy foods like breads and noodles require ingredients that are compatible with hydration, rheology, gas holding, texture development, and heating/cooking procedures. A protein source shown to perform well in one matrix should not be expected to exhibit similar application potential in another system solely because it offers a similar amount of protein (Arshad P. et al., 2025; Caldeira et al., 2024; Ma et al., 2022; Nunes et al., 2020; Seephua et al., 2025).

Microalgal ingredients also present another hurdle to commercial feasibility in that production, harvesting, dewatering, cell disruption, compound extraction, pigment management, flavor management, and drying all increase downstream cost and process complexity. Near-term food applications are therefore likely to be more realistic if microalgae can fulfill several functional roles at once (protein enrichment, natural color, bioactive contribution) instead of being used as low-cost alternatives to purified bulk protein. Commercial success will hinge upon energy-efficient protein recovery processes that can control for unwanted pigment and flavor changes, fulfill a targeted and appropriately-designed matrix, and adhere to regulatory criteria set forth by the final product type (Ali et al., 2025; Braga-Souto et al., 2025; Le et al., 2025).

Protein blending also allows for complementary strategies to be applied, but generated value will always be matrix dependent. Improvements to nutrient profile would require verified amino acid and digestible amino acid data; improvements to techno-functional performance would require blended proteins to exhibit different but complementary functions (structuring, emulsification, foaming, dispersion stabilization); and lastly, sensory attributes can be balanced to account for intensely-flavored or pigmented ingredients when incorporated into a neutral protein background. Though there is limited experimental and comparative data suggesting that blends of plant-proteins can outperform their individual components from a nutritional or techno-functional perspective, protein synergy should never be presumed if only complementary sources are used (Duque-Estrada et al., 2023; Etzbach et al., 2025; Jiménez-Munoz et al., 2021). For example, Duque-Estrada et al. (2023) observed that extrusion cooking allowed for improved nutritional value in blended protein meals through amino acid complementation and processing-related modification of antinutritional factors. Simply put, a blend should only be considered beneficial if it performs better than its individual ingredients or an appropriate expected reference under the defined conditions of formulation and processing.

Table 4 presents a summary of condition-defined food-matrix evidence including segregation of directly observed response, matrix-level interpretation, and remaining validation requirements. Selection of ingredients should be dictated first by technological functionality within the target food matrix and subsequently by material form, analytical definition, nutritional/digestibility evidence, structure-function relationship, sensory and safety limitations, regulatory considerations, and direct evidence of matrix performance. Modification, blending, or reformulation should be evaluated experimentally where a single ingredient does not meet all food-matrix requirements rather than expected to inherently yield improved matrix functionality.

## 6 Safety-related chemical and regulatory considerations

Suitability also encompasses safety and regulatory status of ingredients. Each of these factors is source- and product-form-specific. They should be assessed during ingredient selection and process design and not as an afterthought once formulation has occurred.

Residual antinutritional compounds, off-flavors, and allergenicity (in predisposed individuals) are among some of the most important food-use limitations for lentil and chickpea. Phytate, tannins, and protease inhibitors can impact nutrient availability or digestibility of protein whereas beany or green notes can impact acceptability if not removed/reduced during processing (Saffarionpour, 2024). Chickpea and lentil are also identified sources of allergens for sensitive individuals and therefore their use in novel or increased applications (i.e. concentrated protein ingredients) should not be considered inherently safe without proper study (Abu Risha et al., 2024). While some of the previously mentioned flavor-related limitations can be reduced with processing steps such as extraction (Lakshmipathy et al., 2026), the ingredient should still be evaluated in its intended food application and not treated as though it is sensorially neutral (Ma et al., 2022).

Hazards associated with microalgal ingredients can vary based on the species, cultivation system, form of biomass, and processing history. Commercial *Arthrospira* products marketed as spirulina may be contaminated by toxin-producing cyanobacteria. Thus, microcystins and any other cyanotoxins of potential concern should be controlled and monitored for in spirulina products even when *Arthrospira* itself is not considered a major microcystin producer (U.S. Food and Drug Administration, 2026; Papadimitriou et al., 2021). Nucleic acids are another whole microalgal biomass compositional aspect worth consideration because purine catabolism contributes to uric-acid production. Becker (2007) described nucleic acids as components of the non-protein nitrogen fraction of microalgae that can bias estimates of protein when total nitrogen is multiplied by a fixed conversion factor to calculate protein content. More relevant to commercial products are recent measurements that provide some quantitative context. Muys et al. (2019) found average nucleic-acid contents of 1.4 ± 0.8% DW in commercially available *Chlorella* and 1.6 ± 0.4% DW in Spirulina products. In contrast, older commercial samples described in the same study had closer to 5.4% DW in *Chlorella* and 4.8–5.7% DW in Spirulina. For reference, historically cited Protein Advisory Group guidance in conjunction with Food and Agriculture Organization of the United Nations (FAO), World Health Organization (WHO), and United Nations Children's Fund (UNICEF) limits contributed nucleic-acid load from unconventional protein or single-cell protein sources to about 2 g/day. Using the reported commercial-product data, Muys et al. (2019) calculated what amount of biomass would correspond with this value at ≈73–425 g/day for *Chlorella* and 106–265 g/day for Spirulina products depending on the product. These examples are summarized here to provide general context for when to consider nucleic-acid exposure from microalgal products and are not intended as suggested intake levels nor are they currently legally established safety limits. Nucleic-acid levels should also not be extrapolated between whole biomass and protein-rich extracts or purified isolates without direct analysis (Becker, 2007; Muys et al., 2019). Similar caution should be used for vitamin B12. Although products containing spirulina-type biomass are often marketed for their B12 content, spirulina itself may contain pseudo-cobalamin or other inactive corrinoids. Products should not be presented as reliable B12 sources unless active cobalamin is analytically distinguished (van den Oever & Mayer, 2022; Watanabe et al., 1999).

Duckweed food-grade production must be differentiated from production intended for wastewater treatment or environmental remediation purposes. Nutrients and contaminants from the growth medium can be taken up by Lemnaceae. Depending on water quality, nutrients added, species and production factors, harvested biomass composition and safety can vary. Therefore, biomass from remediation-focused systems should not be presumed safe for human consumption without appropriate controls in place to ensure food-production standards are met, contaminants are monitored for, microbiological safety is assessed, and appropriate government regulations are met (Devlamynck et al., 2020; EFSA NDA Panel, 2021; EFSA NDA Panel, 2022).

Species- and product-form-specific reporting and considerations should also be made for oxalates. The EFSA NDA Panel (2021) discussed a proposed product specification of less than 6500 mg/kg of oxalic acid in their evaluation of *Wolffia globosa* powder. Though this proposed level would correspond to a hypothetical intake of less than 130 mg of oxalic acid from a 20 g intake of the powder, this does not mean there is a blanket safe level of intake from any ingredient. Nor does this mean the product discussed was approved. Instead, exposures should be discussed with the composition of the biomass in question, proposed level in food, and other dietary sources considered. Source-specific safety concerns, chemical or biological drivers, and related analytical and regulatory control considerations are summarized in Table 5.

**Table 5:** Source-specific safety issues, chemical/biological basis, and control considerations for microalgal and duckweed protein ingredients.

Source or ingredient group	Safety issue	Chemical/biological basis and food-use relevance	Analytical or regulatory control needed	Key interpretation limit	Ref.
*Arthrospira*/spirulina products	Microcystins and other cyanotoxins	Commercial spirulina can be contaminated by toxin-producing cyanobacteria, particularly in open cultivation systems; the hazard therefore arises from production-system contamination rather than assuming *Arthrospira* itself is a major microcystin producer	Routine testing for microcystins and relevant cyanotoxins; compliance with product-specific limits/specifications; for U.S. spirulina extract color additive, a negative microcystin toxin result is required	Absence of toxins in one batch does not guarantee absence across suppliers, seasons, or production systems	Papadimitriou et al. (2021); U.S. Food and Drug Administration (2026)
*Arthrospira*/spirulina products	Pseudo-cobalamin and inactive corrinoids	Total corrinoid or microbiological vitamin B12 assays may include biologically inactive analogues and therefore overestimate nutritionally active cobalamin	Distinguish active cobalamin from pseudo-cobalamin using validated analytical methods before making vitamin B12 claims	*Spirulina* should not be described as a reliable vitamin B12 source unless active cobalamin is specifically confirmed	van den Oever and Mayer (2022); Watanabe et al. (1999)
Microalgal whole biomass	Nucleic acid and purine load	Commercial biomass contained 1.4 ± 0.8% DW nucleic acids in *Chlorella* and 1.6 ± 0.4% DW in *Spirulina* in Muys et al. (2019), with higher historical values reported. Purine degradation contributes to uric-acid formation and therefore becomes relevant at sufficiently high exposure	Measure nucleic-acid content in the actual biomass/ingredient and assess exposure at intended daily use. Historical FAO/WHO/UNICEF Protein Advisory Group guidance of ∼2 g/day additional nucleic acids should be treated as contextual rather than a current universal legal limit	Muys et al. (2019) estimated that 2 g nucleic acids corresponded to ∼73–425 g/day *Chlorella* biomass and 106–265 g/day *Spirulina* biomass across tested products. Whole biomass, protein-rich extracts, and purified isolates should not be assumed to have the same nucleic-acid burden	Becker (2007); Muys et al. (2019)
Duckweed/Lemnaceae	Heavy-metal accumulation	Duckweed can absorb dissolved metals from the cultivation medium; contaminant burden therefore depends directly on water quality and production conditions	Batch testing for lead, cadmium, mercury, arsenic, and other relevant metals; compare with applicable food-category limits	Biomass grown for remediation or wastewater treatment is not equivalent to food-grade biomass	EFSA NDA Panel (2022); European Commission (2024)
Duckweed/Lemnaceae	Nitrate accumulation	Nitrate accumulation is influenced by nutrient-medium composition, species, pH, and cultivation conditions; nutrient-rich or recycled water can therefore increase the nitrate burden of harvested biomass	Monitor nitrate/nitrite, especially when nutrient-rich, aquaculture, agricultural, or recycled-water media are used	Nitrate concentration is cultivation-system-specific and should not be generalized across Lemnaceae products	Devlamynck et al. (2020); European Commission (2024)
Duckweed/Lemnaceae	Oxalate content	In the EFSA evaluation of *W. globosa* powder, the proposed oxalic-acid specification was <6500 mg/kg; at that concentration, 20 g powder would correspond to <130 mg oxalic acid. Exposure therefore depends jointly on ingredient concentration and intended intake	Measure oxalate in the specific biomass/product and assess exposure according to intended use level and other dietary sources	The <6500 mg/kg value was product-specific and is neither a universal safe concentration for Lemnaceae nor a general dietary oxalate limit	EFSA NDA Panel (2021)
Duckweed/Lemnaceae	Microbial and cyanobacterial contamination	Aquatic cultivation can introduce microbial hazards or cyanobacterial contaminants through the water source and production environment	Control water source, hygiene, harvesting, washing, drying, and microbiological specifications	Food-grade cultivation must be separated from environmental-remediation applications and assessed under food-production controls	EFSA NDA Panel (2022)

**Note:** Maximum levels and monitoring requirements depend on jurisdiction, product category, final food matrix, and whether the ingredient is marketed as fresh biomass, dried powder, extract, protein concentrate, isolate, food supplement, or color additive. Regulatory limits should therefore be verified for the specific product form rather than generalized at the level of “microalgae” or “duckweed”. DW, dry weight; EFSA, European Food Safety Authority; FAO, Food and Agriculture Organization of the United Nations; NDA, Nutrition, Novel Foods and Food Allergens; UNICEF, United Nations Children's Fund; WHO, World Health Organization.

Setting limits on a product-specific basis allows more robust guidance than general statements about contaminant levels. Title 21 of the Code of Federal Regulations (21 CFR) § 73.530 establishes acceptable levels of contaminants for spirulina extract sold as a color additive in the United States (lead ≤2 mg/kg, arsenic ≤2 mg/kg, mercury ≤1 mg/kg and requirement for no detectable microcystin toxin) (U.S. Food and Drug Administration, 2026). Other contaminant limits depend on the intended food category and jurisdiction (European Commission, 2023b). Protein concentrate produced from *Lemna gibba* and *Lemna minor* and authorized in the European Union (EU) has specifications defined in the novel food authorization that include strict thresholds for cyanotoxins (microcystins/nodularin <0.19 mg/kg) and nitrate (< 3000 mg/kg) (European Commission (2024)), while heavy metal levels must comply with the general limits established for intended food categories (European Commission, 2023b).

State of regulation also needs to be considered on a species and product form basis. In the EU, lentil and chickpea are considered conventional crops with established histories of food consumption; however, regulatory interpretation of protein ingredients made from these species remains product- and process-specific. *Arthrospira platensis* and *Chlorella* have history as food ingredients, while substantially modified or enriched fractions may require product-specific assessment. Dried *Tetraselmis chuii* is authorized as a novel food, while various product forms of *Wolffia* include authorized traditional foods (fresh *Wolffia arrhiza* and/or *Wolffia globosa*) and separately assessed novel foods (*W. globosa* powder) (Araújo & Peteiro, 2021; EFSA NDA Panel, 2021; European Commission, 2014; European Commission, 2021; European Commission, 2023a). Table 6 summarizes the source- and product-form-specific regulatory status within the EU used in this review. Regulatory pathways for these ingredients also differ internationally: Use in the U.S. depends on intended use and resultant regulatory category, Singapore applies pre-market approval to novel foods without a significant history of safe consumption, and Australia/New Zealand require novel foods be assessed and approved before use under the Food Standards Code (U.S. Food and Drug Administration, 2026; Food Standards Australia New, 2026; Singapore Food, 2026). Approval for one product form or jurisdiction should not be assumed for another protein concentrate, isolate, extract, or high inclusion food product.

**Table 6:** EU regulatory status, regulatory triggers, and product-form-specific interpretation of lentil, chickpea, microalgal, and duckweed protein sources relevant to this review.

Protein source or species	EU status/regulatory trigger relevant to this review	Product-form-specific food-use interpretation	Key source
Lentil/lentil protein	Lentil is a conventional food crop with a long history of consumption. In an EU Article 4 consultation, a lentil protein ingredient containing at least 85% protein and produced from red and yellow lentil seeds using standard traditional processing techniques was assessed as not novel	Conventional lentil-derived ingredients should be distinguished from aquatic protein ingredients subject to species- and product-form-specific novel-food assessments. Regulatory evaluation should account for ingredient identity, processing history, allergenicity, and the applicable conditions of food use	European Commission (2023a)
Chickpea/chickpea-derived ingredients	Chickpea is a conventional food crop with a documented history of food use in the EU. The regulatory status of chickpea-derived fractions should be interpreted according to product form and whether processing results in a substantially modified ingredient	Conventional chickpea-derived food ingredients should be distinguished from novel aquatic biomasses, while substantially modified fractions require product-specific regulatory assessment	European Commission (2017)
*Arthrospira platensis*/spirulina	*Arthrospira platensis* biomass has a recognized history of food use in the EU and is not novel. However, modified or enriched forms may require product-specific novel-food assessment; silicon-enriched spirulina was specifically concluded to be a novel food	Conventional spray-dried spirulina powder should be legally distinguished from processed extracts, enriched fractions, or isolated proteins, which may trigger product-specific novel food assessments	European Commission (2025)
*Chlorella* spp.	*Chlorella vulgaris* and *Auxenochlorella pyrenoidosa (*formerly *Chlorella pyrenoidosa)* have documented histories of use in the EU as food or food supplements. However, cryptic taxonomy in commercial strains requires precise genetic validation, and the not-novel status of conventional biomass should not automatically be extrapolated to highly purified, compositionally modified, or substantially different extracts or protein fractions	Whole disrupted *Chlorella* biomass is conventional, but this status should not be extrapolated to highly purified protein isolates or chemical extracts without verifying strain- and process-specific authorization	Araújo and Peteiro (2021); European Commission (2022)
Dried *Tetraselmis chuii*	Authorized as a Novel Food under Regulation (EC) No 258/97 with highly specified food-use categories and strict maximum incorporation limits, including a maximum of 250 mg/day for supplements	Relevant to the technological trials of Nunes et al. (2020) and Qazi et al. (2021); experimental high-inclusion use, e.g., 12% in bread, must be discussed as a processing evaluation rather than an authorized commercial limit	European Commission (2014); European Commission (2017)
*Wolffia globosa* powder	EFSA concluded in 2021 that the additional manganese intake associated with the proposed uses was a safety concern and that the safety of *Wolffia globosa* powder could not be established	Provides a prime example of product-form-specific regulatory concern; drying and concentrating whole *Wolffia* without adequate control of growth-medium trace metals illustrates a product-form-specific regulatory constraint	EFSA NDA Panel (2021)
Fresh *Wolffia arrhiza* and/or *Wolffia globosa* plants	Authorized in the EU as a Traditional Food from a third country under Commission Implementing Regulation (EU) 2021/2191 for fresh *Wolffia arrhiza* and/or *Wolffia globosa* plants as such, subject to defined compositional, contaminant, and microbiological specifications	Authorization of fresh/minimally processed plants is restricted to their whole-cell form; this does not automatically cover dried powders, protein concentrates, or isolated protein fractions	European Commission (2021)
Protein concentrate from *Lemna gibba* and *Lemna minor*	Authorized as a Novel Food under Commission Implementing Regulation (EU) 2024/1048, with specific maximum use levels: 10% in cereal bars, 1.7% in bread, 20% in drink mixes, 6% in noodles, and 1 g/day in supplements for adults	Demonstrates that water-lentil protein can be commercialized as a processed protein ingredient when the authorized production process-including mechanical separation, acidic precipitation, pasteurisation, and spray drying-and specified conditions of use are satisfied	European Commission (2024)

**Note:** The EU Novel Food Catalogue is a non-binding orientation tool and should be read together with the Union list of authorized novel foods. Regulatory status depends on species, product form, processing history, intended food category, and authorized conditions of use. Therefore, whole biomass, dried powder, extract, protein concentrate, isolate, and modified or enriched ingredient forms should not be treated as legally equivalent (Araújo & Peteiro, 2021; European Commission, 2017). EC, European Community; EFSA, European Food Safety Authority; EU, European Union; NDA, Nutrition, Novel Foods and Food Allergens.

## 7 Cross-source discussion and critical synthesis

The matrix of evidence presented above demonstrates that apparent differences between lentil, chickpea, microalgal, and duckweed proteins cannot be adequately interpreted by source identity alone. From composition to digestibility, processing, functionality, food application, safety and regulation: the strength of an apparent cross-source difference depends on material form, analytical basis, processing history, assay conditions, and maturity of available evidence. The following discussion will therefore focus on three cross-cutting themes: analytical comparability/evidence maturity; processing-structure-function trade-offs/conflicting evidence; and translational-readiness for food application.

### 7.1 Evidence maturity and analytical comparability

Comparing sources, it becomes clear that what separates these four protein groups is not merely purported protein content, but evidence maturity and comparability. Evidence for lentil and chickpea protein more commonly targets defined flour, concentrate, isolate, or cooked food materials, and utilizes established analytical and protein-quality approaches. Evidence for microalgal and duckweed protein products, however, describes whole biomass, disrupted biomass, extracts, concentrates, and isolates; compositional and functional differences can therefore be affected by material form (Appenroth et al., 2017; Devlamynck et al., 2020; Higa et al., 2024; Lourenço et al., 2004). This consideration is particularly important when discussing estimated nitrogen-derived protein. Reported nitrogen-to-protein conversion factors within the published microalgae evidence varied experimentally from 2.53 to 5.77, demonstrating that N × 6.25 cannot be assumed to provide equivalent estimates for these species across all stages of cultivation or forms of biomass (Lourenço et al., 2004). Reported protein content within the duckweed scientific literature is also affected by nitrogen method and conversion factor, cultivation condition, and basis of reporting (Appenroth et al., 2017; Devlamynck et al., 2020).

An analogous situation exists for protein-quality evidence. Lentil and chickpea protein quality has been characterized using fecal-protein-based DIAAS-type assessments and, for cooked chickpea, through direct measurement of true ileal amino-acid digestibility (Han et al., 2020; Nosworthy et al., 2018, Nosworthy et al., 2020). In contrast, much of the evidence characterizing microalgal and duckweed protein quality has relied on in vitro digestion, protein accessibility, extraction-dependent release, or calculated scores that may use externally derived digestibility coefficients (Dhamaratana et al., 2025; Egger et al., 2026; Pauliukaitytė et al., 2026; Qazi et al., 2021). Therefore, these metrics imply that numerical values produced by these techniques should not be placed on the same interpretive level. Ultimately, when data sources are not sufficiently aligned in terms of materials, analytical method, processing history, and digestibility metrics, the more defensible conclusion that can be drawn when comparing protein sources is that the maturity of the evidence supporting them differs, not their innate nutritional value.

### 7.2 Processing-structure-function trade-offs and conflicting evidence

Supporting evidence can also be found by considering how increased treatment intensity often fails to produce a consistently improved protein ingredient. Though high-pressure homogenization reduced particle size and increased surface hydrophobicity of lentil protein isolate while also improving solubility and dispersion stability, the solubility response was not strictly monotonic as pressure increased (Malterre et al., 2025). Controlled enzymatic hydrolysis of *Wolffia globosa* increased solubility and improved foaming behavior at specific degrees of hydrolysis while oil-binding capacity and emulsion stability were reduced at higher levels of hydrolysis (Siriwat et al., 2023). These findings illustrate how processing-induced structural changes may improve one functional endpoint but negatively impact another depending on the type and extent of processing (Ma et al., 2022; Nikbakht Nasrabadi et al., 2021). Because of this, processing should be optimized for functionality of interest in the intended application rather than towards a singular maximal response.

Of course, apparent differences among processing routes will need to be decoupled from differences in ingredient identity. The commercial data De Angelis et al. (2024) compared yielded significantly different pooled rheological responses between dry fractionated and wet extracted ingredients, yet individual values overlapped, while the groups differed in botanical source, supplier, protein content, and residual matrix. The data therefore suggest an association within the tested ingredient set but did not demonstrate fractionation route alone imparts a generalizable performance difference. Similar warnings can be applied to any scenario where pH-dependent charge, hydrophobicity, molecular-weight distribution, or other structural indicators shift concurrently with changes in functionality. A mechanistic claim can only be as strong as the experiments used to support it; it is strongest when processing treatments, measured structural mediators, and functional endpoints are measured within the same defined material using an appropriate untreated control. If this is not possible, then the relationship should be considered an experimentally supported association rather than a mechanism that has been independently demonstrated to be causal.

One final form of source-level asymmetry concerns post-extraction modification. Greater diversity of controlled mechanical, enzymatic, thermal, fermentation, and extrusion treatments has been investigated across the reviewed pulse-protein literature than is seen within microalgal studies, which have remained focused on recovery/enrichment, and duckweed literature has provided limited direct evidence for effects of post-extraction modification at all (Le et al., 2025; Malterre et al., 2024, Malterre et al., 2025; Siriwat et al., 2023; Suarez Garcia et al., 2018). This should not be read to imply that aquatic proteins are any less modifiable than land-based proteins. Instead, it reflects a gap in the available direct evidence that limits equivalent cross-source assessment of modification strategies with current literature.

### 7.3 Translational readiness and research priorities

Analytical maturity at laboratory scale does not always equate to readiness for application. Lentil/chickpea have a comparatively strong database of food-matrix studies, yet concerns over off-flavors, allergenicity, antinutritional factors, and loss of functionality due to processing still remain (Abu Risha et al., 2024; Arshad P. et al., 2025; Caldeira et al., 2024; Saffarionpour, 2024). Microalgal proteins offer high protein density and can offer multifunctional biomass, but there is increased complexity to contend with including cultivation/harvesting methods, cell disruption methods, pigment/flavor composition, drying technologies, and regulatory status (Ali et al., 2025; Braga-Souto et al., 2025; Le et al., 2025). Duckweed is somewhere in the middle: there are emerging food-use evidence bases yet understanding species identity, cultivation-water quality, contaminant control, desired product form, and intended regulatory classification are all key factors (EFSA NDA Panel, 2021, EFSA NDA Panel, 2022; Sulaiman et al., 2025). These examples highlight that readiness for translation should be measured across multiple parameters instead of ranking protein sources. Key considerations should include analytical certainty, process repeatability, in-food performance, sensory acceptance, safety, scalability, and intended regulatory status.

Sensory quality and scalability are important translational hurdles that remain even when safety requirements are met. Legume proteins often require the management of beany, green, bitter or other lingering flavor notes; microalgae may contribute strong color, grassy/marine flavors, bitterness, and persistent aftertaste; and duckweed may have issues with green color and grassy flavor (Braga-Souto et al., 2025; Saffarionpour, 2024; Sulaiman et al., 2025). For microalgae, cost of cultivation, harvesting, cell disruption, pigment/flavor management, drying, and batch consistency can also restrict scalability if the purified protein is meant to compete with established commodity proteins (Ali et al., 2025; Le et al., 2025).

Reflecting on the described comparability limitations, five concrete measurable recommendations are made for future research priorities. First, compositional studies should report species, ingredient form, moisture basis, protein-determination method, nitrogen-to-protein conversion factor, amino acid method, protein purity, recovery yield, limiting indispensable amino acids, and relevant non-protein components. Second, digestibility studies should differentiate between IVPD, standardized INFOGEST digestion, total or amino-acid-specific digestibility, PDCAAS, and DIAAS values; comparisons between sources should match ingredient forms and histories if intended (Brodkorb et al., 2019; Egger et al., 2026). Employing matched INFOGEST/INFOGEST Quant conditions for digestion phases, pH and enzyme activities, bile concentration, digestion time, sample handling, and endpoint definitions across matched ingredient forms would reduce protocol-driven variability and improve digestibility comparisons between sources (Brodkorb et al., 2019; Egger et al., 2026).

Third, assay of structure-function relationships should use matched assay conditions and quantify relevant structural and functional endpoints reported. This includes particle size, surface hydrophobicity, ζ-potential, molecular-weight distribution, solubility, emulsification/foaming capacity and stability, gelation, and any other relevant assay conditions. Fourth, application reporting should include incorporation level, matrix composition, processing conditions, physical stability metrics, texture/rheology, color, sensory response, and storage stability. Proposed protein-blend synergies should be demonstrated relative to the individual ingredients or another appropriate expected reference when conditioned under identical formulation parameters (Duque-Estrada et al., 2023; Etzbach et al., 2025). Fifth, translational studies should report on pilot-scale recovery, water & energy demand, drying requirement, throughput, batch reproducibility, compliance with safety thresholds of relevant contaminants, sensory stability, estimated ingredient cost, and readiness for application. Reported together, these data would allow for more easily reproducible comparisons and design of condition-defined protein blends and food matrices.

## 8 Conclusions

Compared data sets among lentil, chickpea, microalgal, and duckweed proteins should be qualified by analytical characterization conditions to be meaningful. Protein content, amino acid composition, digestibility, and techno-functional performance cannot be compared reliably without specifying material form, protein-determination method, nitrogen-to-protein conversion factor, amino acid analytical method, digestibility metric, processing history, and assay conditions. Generally, food-application evidence is more extensive for lentil and chickpea than microalgal and duckweed proteins. Again, this speaks to evidence that is available in the literature and should not be taken as innate disparities between these protein sources in food matrices. Similar qualifications regarding biomass form, cell disruption/extraction history, presence of non-protein N, sensory limitations, contaminants and their control, food-grade production methods, and product-form-specific regulatory status also apply for microalgae and duckweed proteins based on species identity.

Therefore, informing future food applications is less about ranking protein sources and more about designing protein systems under defined conditions. Standardized primary research is needed that utilizes verified methods of protein and amino acid analysis, clearly differentiated measures of digestibility, quantitative evaluations of structure-function relationships, food-matrix validation, and pilot-scale studies. Protein blending may be beneficial, but nutrition or techno-functional synergy should be demonstrated under defined formulation and processing conditions rather than expected based on protein source identity.

## CRediT authorship contribution statement

**Duy H. Truong:** Conceptualization, Methodology, Data curation, Writing – original draft, Writing – review & editing, Supervision, Project administration. **Chi Hao Nguyen:** Conceptualization, Methodology, Investigation, Data curation, Writing – original draft, Writing – review & editing.

## Declaration of generative AI and AI-assisted technologies in the writing process

ChatGPT from OpenAI was used for language, grammar, and readability during manuscript preparation. The tool was not used to generate original scientific data, perform data analysis, replace scientific interpretation, or determine the conclusions of the review. All AI-assisted edits were reviewed, edited, verified, and approved by the authors, who take full responsibility for the content of the published article.

## Funding

This research did not receive any specific grant from funding agencies in the public, commercial, or not-for-profit sectors.

## Declaration of competing interest

The authors declare that they have no known competing financial interests or personal relationships that could have appeared to influence the work reported in this paper.

## Data Availability

No data was used for the research described in the article.

## Appendix A Supplementary data

Supplementary material: Supplementary Table S1 summarizes the effects, technological benefits, and limitations of processing and modification methods applied to alternative proteins, while Supplementary Table S2 presents the search strategies used for literature identification.
