# Supplementary material for: Lentil, chickpea, microalgae, and duckweed proteins in plant-based foods: a food chemistry perspective on composition, processing, functionality, and digestibility
Source: Food Chem X. 2026 Nov 5;40:104744. doi: 10.1016/j.fochx.2026.104744 (PMC13770633; doi:10.1016/j.fochx.2026.104744)
Supplement: Supplementary file 1 [file mmc1.docx]

**Supplementary Material**

**Supplementary Table S1.** Effects of processing and modification methods on the techno-functional properties of alternative proteins

| **Processing/**  **modification method** | **Main mechanism** | **Typical effects on proteins** | **Technological benefits** | **Limitations/risks** |
| --- | --- | --- | --- | --- |
| Dry fractionation | Protein enrichment through mechanical or air-classification-based separation | Causes less denaturation and better preserves a near-native protein state (De Angelis et al., 2024; Schutyser et al., 2025) | Clean-label process, low water use, and sustainability advantages | Generally lower purity; functional properties may be variable |
| Alkaline wet extraction-isoelectric precipitation | Protein solubilization followed by selective precipitation | Increases protein purity but may induce substantial denaturation (De Angelis et al., 2024; Grasso et al., 2022; Le et al., 2025) | Enables better ingredient standardization and is suitable for producing isolates | High water consumption; may reduce solubility or cause unfavorable structural changes |
| Ultrasound/ high-shear treatment | Disruption of aggregates and reduction of particle size | Improves dispersion and increases surface area (Duangjarus et al., 2022; Malterre et al., 2024; Nitiwuttithorn et al., 2024) | Enhances solubility, system stability, and emulsification | Excessive treatment may cause undesirable protein denaturation |
| Enzymatic hydrolysis | Controlled cleavage of peptide bonds | Reduces molecular size and increases solubility (Gasparre et al., 2025; Pauliukaitytė et al., 2026) | Useful for beverages and emulsion systems; may improve digestibility | May generate bitterness or reduce gel-forming ability if hydrolysis is excessive |
| Fermentation | Biological modification of proteins and co-existing compounds | May reduce antinutritional factors and off-flavors (Yousefi et al., 2025) | Can improve sensory quality, digestibility, and, in some cases, functionality | Difficult to control consistently at large scale |
| Heat treatment | Protein unfolding and denaturation | Enhances protein-protein interactions and supports gel formation (Hülsebusch et al., 2025; Nikbakht Nasrabadi et al., 2021) | Beneficial for structuring and water retention | Excessive treatment can reduce solubility and increase aggregation |
| High-moisture extrusion | Heat, moisture, and shear-driven structuring | Rearranges proteins into fibrous structures (Hülsebusch et al., 2025; Sha & Xiong, 2020; Zang et al., 2025) | Highly important for meat analogue production | May promote oxidation and alter nutritional value |
| Delipidation/ decolorization | Removal of lipids, pigments, and odor-active compounds | Increases purity and improves sensory quality (Braga-Souto et al., 2025; Le et al., 2025; Mosibo et al., 2024) | Particularly important for microalgae | May increase processing costs and reduce recovery yield |

Note: The processing and modification approaches summarized here are not equally represented across the four protein-source groups. Within the primary studies retained in this review, microalgal evidence is dominated by cell disruption, recovery, and enrichment, whereas direct post-extraction modification evidence remains limited. For duckweed, direct evidence is primarily represented by controlled enzymatic hydrolysis.

**Supplementary Table S2**. Search strategy used for literature identification

| **Search theme** | **Boolean search string** |
| --- | --- |
| Lentil | (“lentil protein” OR “Lens culinaris protein”) AND (“plant-based” OR food OR beverage OR bakery OR “meat analogue” OR “functional properties” OR solubility OR emulsification OR foaming OR gelation OR digestibility OR PDCAAS OR DIAAS) |
| Chickpea | (“chickpea protein” OR “Cicer arietinum protein”) AND (“plant-based” OR food OR beverage OR bakery OR “meat analogue” OR “protein isolate” OR fractionation OR solubility OR emulsification OR foaming OR gelation OR digestibility OR PDCAAS OR DIAAS) |
| Microalgae | (microalgae OR microalgal OR Arthrospira OR Spirulina OR Chlorella OR Tetraselmis OR Nannochloropsis) AND (protein OR “protein isolate” OR “protein concentrate” OR extraction OR functionality OR digestibility OR “food application” OR beverage OR bread OR pasta OR “plant-based”) |
| Duckweed | (duckweed OR Lemnaceae OR Lemna OR Wolffia OR Spirodela OR Landoltia) AND (protein OR “protein extract” OR “protein concentrate” OR extraction OR functionality OR digestibility OR “food application” OR “novel food”) |
| Microalgal analytical/ safety issues | (microalgae OR Spirulina OR Arthrospira OR Chlorella) AND (“nitrogen-to-protein conversion factor” OR “non-protein nitrogen” OR nucleic OR purine OR microcystin OR cyanotoxin OR “pseudo-cobalamin” OR “vitamin B12”) |
| Duckweed analytical/ safety issues | (duckweed OR Lemnaceae OR Lemna OR Wolffia) AND (“nitrogen-to-protein conversion factor” OR nitrate OR oxalate OR “heavy metal” OR contaminant OR “food safety” OR “novel food” OR EFSA) |
| Cross-source structure–function and digestibility | (“plant protein” OR “alternative protein”) AND (“structure-function” OR solubility OR emulsification OR foaming OR gelation OR “surface hydrophobicity” OR “zeta potential” OR DSC OR INFOGEST OR PDCAAS OR DIAAS) |
| Targeted ileal-DIAAS verification | (“lentil” OR “Lens culinaris” OR “chickpea” OR “Cicer arietinum”) AND (“DIAAS” OR “digestible indispensable amino acid score” OR “true ileal digestibility” OR “ileal amino acid digestibility”) AND (“pig” OR “rat” OR “cooked”) |

Note: The targeted ileal-DIAAS verification search was conducted on 10 October 2026.
